# The DJ1-Nrf2-STING axis mediates the neuroprotective effects of Withaferin A in Parkinson’s disease

**DOI:** 10.1038/s41418-021-00767-2

**Published:** 2021-03-24

**Authors:** Miao Zhao, Bingwei Wang, Chenyu Zhang, Zhijie Su, Bingbing Guo, Yun Zhao, Ruimao Zheng

**Affiliations:** 1 Department of Anatomy, Histology and Embryology, Health Science Center, Peking University, Beijing, China; 2 Neuroscience Research Institute, Peking University, Beijing, China; 3 Key Laboratory for Neuroscience of Ministry of Education, Peking University, Beijing, China; 4 Key Laboratory for Neuroscience of National Health Commission, Peking University, Beijing, China

**Keywords:** Neurological disorders, Drug development

## Abstract

The pathogenesis of Parkinson’s disease (PD) remains unclear, and there is no disease-modifying agent for PD. Withaferin A (WA), a naturally occurring compound, has emerged as a neuroprotective agent. However, the mechanisms by which WA is neuroprotective in PD are unknown. Here we show that WA protected against loss of dopaminergic neurons, neuroinflammation, and motor deficits in MPTP-induced PD mouse models. Whole-genome deep sequencing analysis combined with Meta-analysis of human PD studies reveal that DJ1, Nrf2, and STING in substantia nigra pars compacta (SNc) are linked to anti-PD effect of WA. We found that WA activated DJ1 and Nrf2, and suppressed STING within SNc; and overexpression of STING in SNc dampened the effect of WA. Using genetically modified mice (DJ1-KO, Nrf2-KO, STING^gt/gt^ and STING-KO) and immunolabeling technique, we identified that WA targeted DJ1-Nrf2-STING pathway in dopaminergic neurons; and we demonstrate that STING might be an important factor in PD pathogenesis. In addition, WA alleviated accumulation of phosphorylated α-synuclein (p-α-syn) and insoluble α-syn within SNc in adeno-associated virus (AAV)-mediated human α-syn overexpression PD model. Our comparative analysis on whole-genome transcriptome profiles suggests that STING might be a key target of WA and amantadine in PD treatment. This study highlights a multifaceted role for WA in neuroprotection, and suggests that WA can be a potential candidate for treatment of PD.

## Introduction

Parkinson’s disease is the second most common neurodegenerative disease, affecting ~1% of the population older than 50, and 2–3% of the population older than 65 years of age [1]. The incidence of PD progressively increases with age, along with a trend of young-onset PD, and it is predicted that the prevalence of PD will reach over 14 million cases by 2040 [2], which points to an urgent need for the development of novel and effective therapeutic agents. The PD etiology remains elusive but is thought to be linked to monogenic mutations in specific genes (<10% of cases) or a poorly understood interplay of genetic and environmental factors, including gene mutations, neurotoxicity, and virus infection [3–5]. The main pathological feature of PD is the loss of dopaminergic neurons in Substantia Nigra compacta (SNc) and their projections to Striatum (STR), accompanying with the accumulation of misfolded α-synuclein (α-syn) in Lewy bodies and Lewy neurites, which result in the motor symptoms: bradykinesia, rigidity and resting tremor [6]. To date, there is no disease-modifying agent for PD patients to slow, halt, or reverse the progression of PD [7].

The naturally occurring compounds, because of their characteristics of the multiple bioactive properties relevant to neuroprotection, are considered to play a promising role in the development of novel agents for PD treatment [8, 9]. Withaferin A (WA), a steroidal lactone with highly lipid solubility, is isolated from the *Withania somnifera*. WA can cross the blood-brain barrier [10]. WA exhibits favorable properties that are neuroprotective, including antioxidant, anti-inflammatory effects; and promotive effect on autophagy and ubiquitin-proteasome pathway (UPS) function [10–12]. The correspondences between WA effects and PD pathogenesis enlightened us to assess whether WA acts as a potential agent for PD treatment.

To investigate the potential mechanisms underlying the neuroprotective role of WA, we performed genome-wide sequencing analysis to inspect the differential expression genes (DEGs) in SNc of WA-treated PD mouse models. We identified a series of target genes that are associated with the neuroprotective effects of WA, such as *DJ1, Nrf2, and TMEM173*. DJ1 (PARK7), a redox-dependent molecular chaperone, acts as a neuroprotective factor [13, 14]. Loss-of function mutations in DJ1 cause early-onset autosomal recessive PD [15]. DJ1 overexpression protects dopaminergic neurons against PD; while DJ1 deficiency displays profound loss of dopaminergic neurons [16]. Nrf2 (nuclear-factor-E2-related factor 2) is a transcription factor. Under the condition of PD-causing toxins, Nrf2 translocates to the nucleus where it binds to antioxidant response element to induce antioxidant and phase II detoxification enzymes, which play neuroprotective roles in PD [17]. Nrf2 gene variant confers a decreased risk and delayed onset of PD in the Swedish and Polish [18]. Nrf2 deficiency exacerbates PD phenotypes [19]. WA mitigates the neuroinflammation by activating Nrf2 [20]. Scopoletin, another naturally occurring compound, acts through DJ1-Nrf2 pathway to mitigate α-syn accumulation [21]. STING (stimulator of interferon genes, TMEM173) is known as an innate immune response regulator, which induces type-I interferon (IFN) signaling to participate in neuronal immunomodulation [22, 23]. The damage-associated molecular patterns (DAMPs) and the pathogen-associated molecular patterns (PAMPs) occur in neurodegenerative diseases (PD or Alzheimer’s disease) [24, 25]. In cytosol, the self-DNA leaked from mitochondria, nucleus, or phagosome serves as DAMPs; while the nucleic acids, such as viral DNA or leaked mitochondrial DNA caused by viral infections serves as PAMPs, which are sensed by cyclic GMP-AMP Synthase (cGAS). The cGAS activates STING to recruit TBK1 and IRF3 to induce type-I IFN gene (IFN1β) and inflammatory factors. DAMPs and PAMPs contribute to the progression of neurodegeneration [23, 26]. The damaged mitochondria release mitochondrial DNA (mtDNA) in SNc of both PD patients and MPTP-received mice, causing STING-mediated neuroinflammation, which deteriorate PD progression [24, 27, 28]. The loss of dopaminergic neurons in SNc, the motor defect and the elevated neuroinflammation are mitigated by loss of STING [24]. Viral infection activates STING, and accelerates the formation of Lewy bodies and the loss of dopaminergic neurons [29, 30]. Amantadine, an antiviral medication, mainly used in treatment of influenza A virus infection, which is also used in treatment of PD [31]; it relieves the PAMPs caused by virus infection [30]. The antiviral therapy reduces the PD incidence in human [32]. Intriguingly, epidemiological data show that influenza A viral infection increases the risk of PD [33]. Although amantadine is believed to promote dopamine release, and it also appears to be a weak N-methyl-D-aspartate (NMDA) receptor antagonist to antagonize the neurotoxic processes [34]; the mechanisms of amantadine in PD treatment are far from clear. Based on these observations, two hypotheses are put forward. First, STING can be activated by DAMPs and PAMPs, and thus causes the loss of SNc dopaminergic neurons, which may be an undiscovered mechanism underlying PD pathogenesis. Second, suppression of STING-mediated neuroinflammation may be involved in neuroprotective effects of WA. Thus, we analyzed the DEGs in SNc of the mice treated with WA, amantadine and other anti-Parkinson’s agents to test our hypotheses. Moreover, to obtain a comprehensive understanding of the neuroprotective effects of WA, we employed the multiple PD models to better model the degenerative features of PD, including the MPTP-induced PD mouse model, the AAV-mediated human α-syn overexpression PD model and the MPP^+^ treated human dopaminergic neurons. The Meta-analysis is widely used to explore the disease pathology, and provides more precise estimate for effect of PD treatment in human [35]; thus, we also performed Meta-analysis to probe the transcriptomic profiles in SNc of PD patients, combined with a sequencing analysis in murine models. Together, our work identifies WA as an important anti-PD agent and proposes a pathway that can be targeted for treatment of PD and related neurodegenerative disorders.

## Results

### WA protects against loss of dopaminergic neurons in MPTP-induced PD mice

**Fig. 1:**
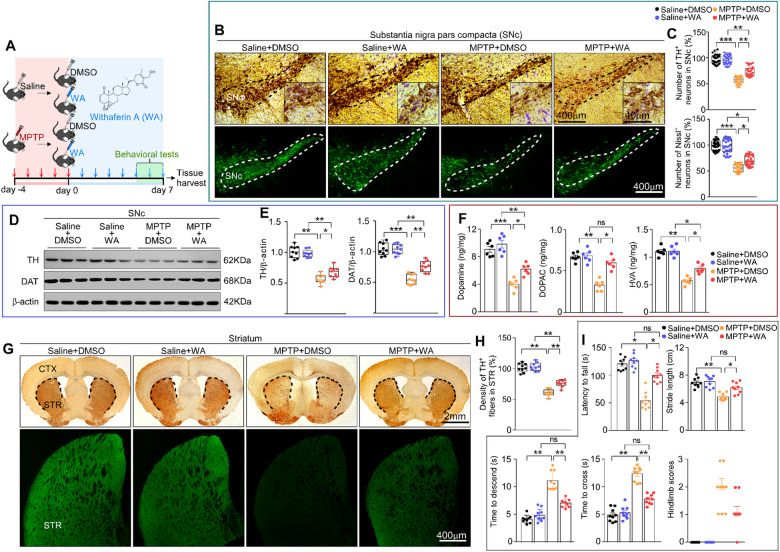
Neuroprotective effects of Withaferin A in MPTP-induced PD mouse model. **A** Diagram of the experimental design. MPTP (20 mg/kg) or vehicle (saline) was injected (i.p.) for 5 consecutive days starting on day -4 after acclimation (3 days), then mice intraperitoneally (i.p.) received WA (20 μg/kg) or vehicle (DMSO) per day for 7 days, tissues were harvested for molecular analyses at day 8 after the last behavior test. **B** Representative photomicrographs of TH and Nissl staining in SNc. Scale bars, 400 μm for low-magnification images and 40 μm for high-magnification images, respectively. **C** Unbiased stereological counts of TH-positive and Nissl-positive neurons in SNc. Data are mean ± s.e.m.; *n* = 30 biologically independent animals; *P = 0.0425 **P = 0.007, ***P < 0.001 by one-way ANOVA with Bonferroni’s post hoc test. **D** Representative immunoblots of TH, DAT, and β-actin in SNc (cropped blot images are shown, see Supplementary Fig. 16 for full immunoblots). **E** Quantification of TH and DAT protein levels in SNc. Data are mean ± s.e.m.; *n* = 9 biologically independent animals. *P < 0.05, **P < 0.01, and ***P < 0.001. **F** HPLC assessment of dopamine concentrations in striatum of vehicle (saline) or MPTP injected mice treated with vehicle or WA. Data are mean ± s.e.m.; *n* = 6 biologically independent animals; *P < 0.05, **P < 0.01, and ***P < 0.001 by one-way ANOVA with Bonferroni’s post hoc test. **G** Representative photomicrographs of TH staining in striatum, scale bar, 2 mm (upper panel) and 400 μm (down panel). **H** Quantification of TH-positive striatal fiber density. Data are mean ± s.e.m.; *n* = 9 biologically independent animals. **I** Time to traverse beam apparatus, time to descend pole, Hind-limb clasping reflex score, fall latency from an accelerating rotarod and gait analysis. Data are mean ± s.e.m.; *n* = 9 biologically independent animals. The one-way ANOVAs were used for statistical analysis followed by Bonferroni’s post hoc test. *P < 0.05, **P < 0.01, and ***P < 0.001. ns, not significant.

The neuroprotective potential of WA was first evaluated in MPTP-induced PD mouse model. WA treatment at the doses of 20, 200, and 2000 μg/kg relieved the loss of dopaminergic neurons and motor deficits (Supplementary Fig. 1. WA (20 μg/kg for 7, 14 or 21 days) protected the dopaminergic neurons and motor deficits (Supplementary Fig. 2). To explore the mechanism underlying the effects of WA, the treatment with WA (20 μg/kg) was selected (Fig. 1A). WA protected against the loss of dopaminergic neurons (~45% loss and ameliorated to 74%) (Fig. 1B, C), relieved the reduction in tyrosine hydroxylase (TH) (69% versus 56%) and dopamine transporter (DAT) (76% versus 54%) immunoreactivity in SNc (Fig. 1D, E). The reductions in dopamine and its metabolites (DOPAC, HVA) were normalized by WA (Fig. 1F). WA led to a greater TH^+^ fibers density (71% versus 58%) in STR in response to MPTP (Fig. 1G, H). Pole descent, rotarod test, beam traversal, hindlimb clasping reflexes and gait test revealed that the impaired motor coordination and balance were mitigated by WA (Fig. 1I). The high immunoreactivity of GFAP and Iba1 was suppressed in SNc and STR of WA-received mice (Supplementary Fig. 3A–F). These results showed that WA mitigates PD-like symptoms, suggesting that WA appears to be a candidate for disease-modifying therapy in PD.

### DJ1, Nrf2, and STING are identified as targets of WA

**Fig. 2:**
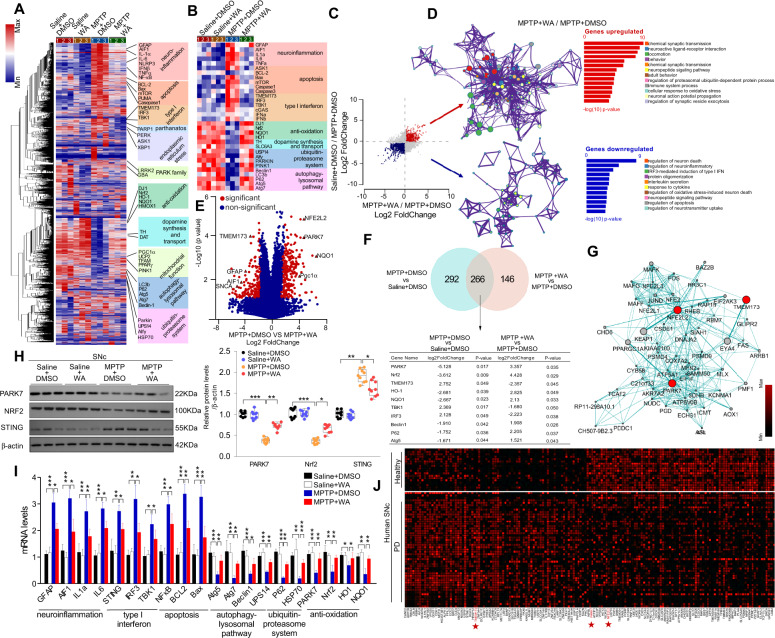
Gene expression analysis of SNc in MPTP-induced PD mice treated with Withaferin A. **A** Hierarchical clustered heatmap of gene expression profiles for WA or vehicle treatment in SNc of MPTP-induced PD mice. **B** Heatmap of DEGs of MPTP-received mice treated with WA or vehicle. **C** Scatter plot highlights the DEGs of WA treatment compared with vehicle in MPTP-recieved mice, upregulated genes are colored in red, downregulated genes are colored in blue. (*P* < 0.05 with unpaired two-tailed Student’s *t* tests). **D** Gene Ontology enrichment was based on DEGs that have a *P* value smaller than 0.05. Enrichment analysis for Gene Ontology terms among the genes of a gene–trait correlation module was performed using Metascape. **E** Volcano plot displays DEGs of WA treatment compared with vehicle in MPTP-received mice. Significantly altered genes are colored in red, insignificantly altered genes are colored in blue. **F** Venn diagram of overlapping significantly changed genes (±1.2 fold, *P* < 0.05). The top ten overlapping genes are presented. **G** Protein–protein interaction network identified among DJ1, Nrf2 and STING using GeneMANIA (direct interaction database). **H** Representative immunoblots of DJ1, Nrf2, STING and β-actin, and quantification of DJ1, Nrf2, STING protein levels in SNc (cropped blot images are shown, see Supplementary Fig. 16 for full immunoblots). Data are mean±s.e.m.; *n* = 9 biologically independent animals; *P < 0.05, **P < 0.01, and ***P < 0.001. **I** Relative mRNA expression of the indicated genes in SNc. Data are mean ± s.e.m.; *n* = 8 biologically independent animals. The one-way ANOVAs were used for statistical analysis followed by Bonferroni’s post hoc test. *P < 0.05, **P < 0.01, and ***P < 0.001. **J** Heatmap of the top 100 significant genes of SNc of human PD samples analyzed by Meta-analysis (FDR < 0.05). Expression values of each gene are standardized within each dataset. Hierarchical clustering was used to cluster samples and genes.

To analyze the mechanism underlying the beneficial effects of WA, whole-genome RNA-sequencing was performed. WA upregulated the genes associated with dopamine synthesis and mitochondrial function in SNc, and caused a mitigation of genes related to anti-oxidative stress; autophagy-lysosome pathway (ALP) and UPS. WA normalized the genes linked to neuroinflammation and apoptosis (Fig. 2A, B). Strikingly, high levels of STING-dependent response genes were observed in SNc of MPTP-treated mice, which was normalized by WA (Fig. 2B). WA induced the pathways associated with dopamine synthesis, mitochondrial function, autophagy and locomotory behavior; while it inhibited the pathways related to inflammation, apoptosis, STING-mediated neuroinflammation and protein oligomerization (Fig. 2C, D). WA led to an induction of DJ1, Nrf2, HO1, and NQO1, and a decrease in STING, TBK1, and IRF3 (Fig. 2E, F, H, I). The network analysis using GeneMANIA predicted a potential interaction and a high clustering coefficient among DJ1, Nrf2, and STING (Fig. 2G). Meta-analysis on genome-wide GEO datasets from 72 patients with PD and 58 healthy controls showed high level of STING, and low levels of DJ1 and Nrf2 in SNc of PD patients (Fig. 2J). Together, these results support that STING pathway may be linked to PD pathogenesis, and DJ1, Nrf2 and STING may be important factors that underlie the pleiotropic effects of WA against PD.

### The neuroprotective effects of WA against PD are DJ1-dependent

**Fig. 3:**
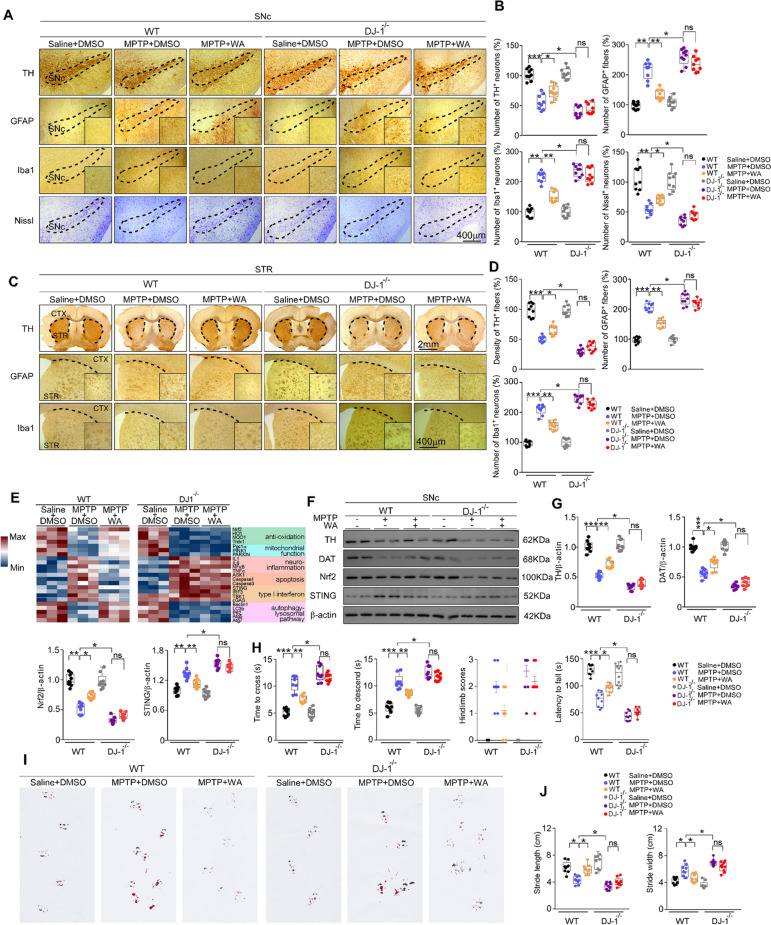
Neuroprotective effects of Withaferin A in PD are DJ1 dependent. **A** Representative TH, GFAP, Iba1 and Nissl staining of SNc in WT and DJ1-KO mice, scale bar, 400 μm. **B** Unbiased stereological counts of TH^+^, GFAP^+^, Iba1^+^, and Nissl^+^ cells in SNc of WT and DJ1-KO mice. Data are mean ± s.e.m.; *n* = 9 biologically independent animals; *P < 0.05, **P < 0.01, and ***P < 0.001 by two-way ANOVAs followed by Tukey’s multiple comparisons test. **C** Representative photomicrographs of TH, GFAP, Iba1 staining in STR of WT and DJ1-KO mice, scale bar, 2 mm. **D** Stereological counts of TH, GFAP, Iba1 positive cells in STR of WT and DJ1-KO mice. Data are mean ± s.e.m.; *n* = 9 biologically independent a*n*imals; *P < 0.05, **P < 0.01, and ***P < 0.001 by two-way ANOVAs followed by Tukey’s multiple comparisons test. **E** Relative mRNA expression in SNc of WT and DJ1-KO mice. Data are mean ± s.e.m.; *n* = 8 biologically independent animals. **F, G** Representative immunoblots of TH, DAT, Nrf2 and STING in SNc (cropped blot images are shown, see Supplementary Fig. 16 for full immunoblots), quantification of TH, DAT, Nrf2, and STING levels. Data are mean ± s.e.m.; *n* = 9 biologically independent animals; *P < 0.05, **P < 0.01, and ***P < 0.001 by two-way ANOVAs followed by Tukey’s multiple comparisons test. **H** Time to traverse beam apparatus, time to descend pole, hind-limb clasping reflex score, fall latency from an accelerating rotarod and (**I** and **J**) gait analysis. Data are mean ± s.e.m.; *n* = 9 biologically independent animals. Two-way ANOVA followed by Tukey’s post hoc test. *P < 0.05, **P < 0.01, and ***P < 0.001. ns, not significant.

To determine whether DJ1 is involved in the neuroprotective role of WA, DJ1-KO mice were employed. WA lost its potency to protect against both of the loss of dopaminergic neurons (55% loss) (Fig. 3A, B) and the decrease of TH^+^ fibers density (58% loss) (Fig. 3C, D) induced by MPTP in DJ1-KO mice. Similarly, WA had little effect on the reduction of TH and DAT immunoreactivity (Fig. 3F, G), and the impairment of motor deficits (Fig. 3H, I). The inhibitory effect of WA on the neuroinflammation related to reactive astrocytes and microglia triggered by MPTP was halted in DJ1-KO mice (Fig. 3A–E). WA increased the immunoreactivity of DJ1 in dopaminergic neurons (Supplementary Figs. 4A and  5B), but not in astrocytes or microglia (Supplementary Figs. 4B and 5A). Similar result was confirmed in human dopaminergic neurons (Supplementary Fig. 12D). The restorative effect of WA on the decreased protein level of Nrf2 (Fig. 3F, G) and the decreased mRNA levels of the genes in the downstream of Nrf2 pathway triggered by MPTP were impaired in DJ1-KO mice (Fig. 3E). Similarly, DJ1 knockdown inhibited the promotive effect of WA on Nrf2 expression in MPP^+^ treated human dopaminergic neurons (Supplementary Fig. 12A–E). The elevated STING expression induced by MPTP was reversed by WA (Fig. 3E–G). WA reduced the activation of STING pathways in SNc treated with MPTP (Fig. 3E). Likewise, WA inhibited the activation of STING pathway triggered by MPP^+^ in human dopaminergic neurons (Supplementary Fig. 12). The analyses of the genes related to mitochondrial function and ALP yielded no overt differences between WA and controls (Fig. 3E). Together, these results raise the tantalizing possibility of DJ1 for anti-PD effects of WA.

### WA exerts neuroprotective effects on dopaminergic neurons via DJ1-Nrf2 axis

**Fig. 4:**
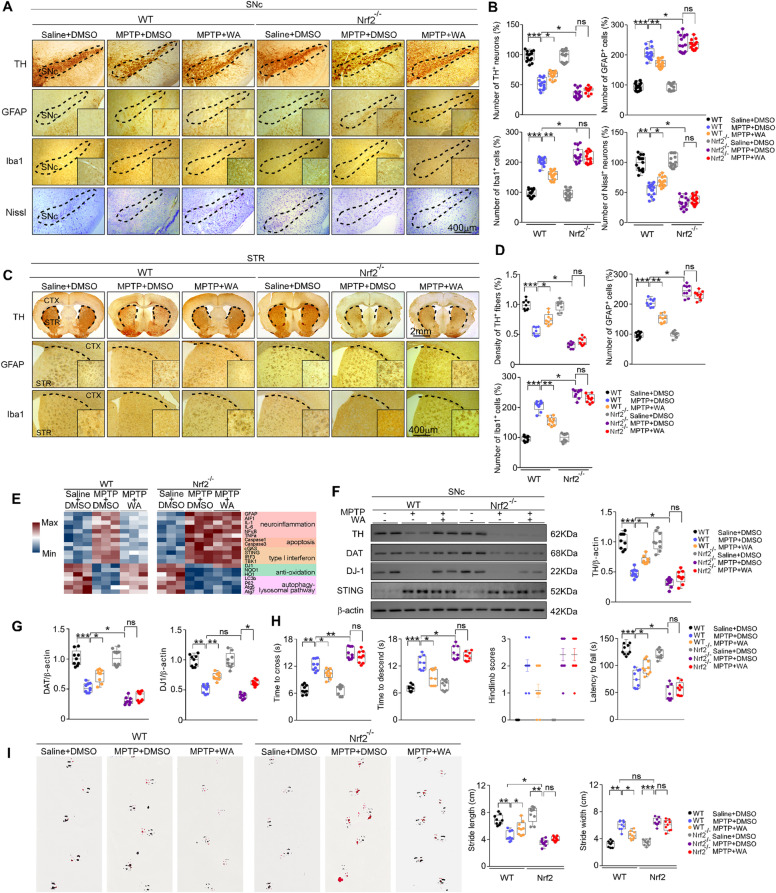
Neuroprotective actions of Withaferin A in PD are mediated by DJ1-Nrf2 axis. **A** Representative TH, GFAP, Iba1 and Nissl staining of SNc in WT and Nrf2-KO mice, scale bar, 400 μm. **B** Unbiased stereological counts of TH^+^, GFAP^+^, Iba1^+^ and Nissl^+^ cells in SNc of WT and Nrf2-KO mice. Data are mean ± s.e.m.; *n* = 15 biologically independent animals; *P < 0.05, **P < 0.01, and ***P < 0.001 by two-way ANOVAs followed by Tukey’s multiple comparisons test. **C** Representative photomicrographs of TH, GFAP and Iba1 staining in STR of WT and Nrf2-KO mice, scale bar, 2 mm. **D** Stereological counts in STR of WT and Nrf2-KO mice. Data are mean ± s.e.m.; *n* = 9 biologically independent animals; *P < 0.05, **P < 0.01, and ***P < 0.001 by two-way ANOVAs followed by Tukey’s multiple comparisons test. **E** Relative mRNA expression in SNc of WT and Nrf2-KO mice. Data are mean ± s.e.m.; *n* = 8 biologically independent animals. **F** Representative immunoblots of TH, DAT, DJ1 and STING in SNc (cropped blot images are shown, see Supplementary Fig. 16 for full immunoblots). **G** Quantification of TH, DAT, DJ1 and STING levels. Data are mean ± s.e.m.; *n* = 9 biologically independent animals. **H** Time to traverse beam apparatus, time to descend pole, Hind-limb clasping reflex score, fall latency from an accelerating rotarod. **I** Gait analysis. Data are mean ± s.e.m.; *n* = 9 biologically independent animals. Two-way ANOVA followed by Tukey’s post hoc test. *P < 0.05, **P < 0.01, and ***P < 0.001. ns, not significant.

To determine whether Nrf2 mediates the effects of WA, Nrf2-KO mice were employed. The Nrf2 deficiency had little effect on the restorative action of WA on DJ1 level in response to MPTP (Fig. 4E, F). Likewise, Nrf2 knockdown by siRNA did not affect the enhanced level of DJ1 induced by WA in MPP^+^ treated human dopaminergic neurons (Supplementary Fig. 12). The neuroprotective effect of WA on the loss of dopaminergic neurons (42% versus 67%) (Fig. 4A, B) and the decrease in TH^+^ fibers density (41% versus 75%) (Fig. 4C, D) was halted in Nrf2-KO mice. WA had little effect on the reduction in TH and DAT immunoreactivity (Fig. 4F, G); and failed to rescue the impaired motor coordination and balance in Nrf2-KO mice (Fig. 4H, I). The mitigative effect of WA on astrocytes, microglia (Fig. 4A–D), and inflammatory factors was dampened in Nrf2-KO mice (Fig. 4E). WA caused a restoration of Nrf2 level mainly in dopaminergic neurons (Supplementary Figs. 6A and 7B). WA also led to an increased Nrf2 nuclear translocation in dopaminergic neuron (Supplementary Fig. 7C). No difference in Nrf2 level was observed after WA treatment in both astrocytes and microglia (Supplementary Fig. 6B, Supplementary Fig. 7A). The mitigative effect of WA on *STING, IRF3, TBK1, TNFα* and *IL6* was impeded in SNc of Nrf2-KO mice (Fig. 4E, F). Similarly, WA failed to suppress STING in MPP^+^ treated human dopaminergic neurons under the condition of Nrf2 knockdown (Supplementary Fig. 12). Nrf2 deficiency impaired the relief of apoptotic genes and the enhancement of ALP-associated genes induced by WA (Fig. 4E). Further, in vitro study showed the high levels of DJ1 and Nrf2 in WA-treated neurons (Supplementary Figs. 4 and 6). These findings suggest that Nrf2, as a central player in the anti-inflammatory and anti-oxidative effects of WA, counterbalances a large number of etiological pathways of PD.

### The effects of WA are mediated by DJ1-Nrf2-STING axis in dopaminergic neurons

**Fig. 5:**
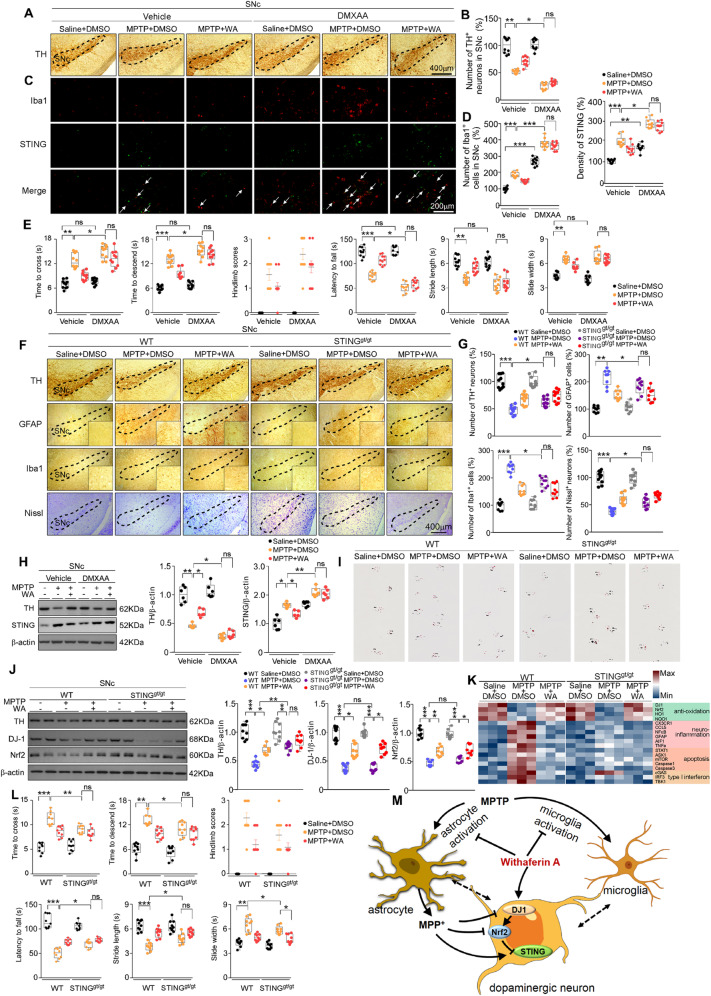
Withaferin A suppresses STING to protect dopaminergic neurons from MPTP neurotoxicity. **A** Representative TH staining of SNc dopaminergic neurons in DMXAA or vehicle-treated mice, scale bar, 400 μm. **B** Unbiased stereological counts of TH^+^ neurons in SNc of DMXAA or vehicle-treated mice. Data are mean ± s.e.m.; *n* = 10 biologically independent animals; *P < 0.05 and **P < 0.01 by two-way ANOVAs followed by Tukey’s multiple comparisons test. **C** Representative Iba1 and STING immunostaining in SNc of DMXAA or vehicle-treated mice, scale bar, 200 μm. **D** Quantification of Iba1 and STING levels. Data are mean ± s.e.m.; *n* = 10 biologically independent animals; *P < 0.05, **P < 0.01, and ***P < 0.001 by two-way ANOVAs followed by Tukey’s multiple comparisons test. **E** Time to traverse beam apparatus, time to descend pole, hind-limb clasping reflex score, fall latency from an accelerating rotarod and gait analysis. Data are mean ± s.e.m.; *n* = 9 biologically independent animals; *P < 0.05, **P < 0.01, and ***P < 0.001 by two-way ANOVAs followed by Tukey’s multiple comparisons test. **F** Representative TH, GFAP, Iba1 and Nissl staining of SNc in WT and STING^gt/gt^ mice, scale bar, 400 μm. **G** Unbiased stereological counts of TH^+^, GFAP^+^, Iba1^+^ and Nissl^+^ cells in SNc of WT and STING^gt/gt^ mice. Data are mean ± s.e.m^.^; *n* = 9 biologically independent animals; *P < 0.05, **P < 0.01, and ***P < 0.001 by two-way ANOVAs followed by Tukey’s multiple comparisons test. **H** Representative immunoblots and quantification of TH and STING in SNc of DMXAA treated mice (cropped blot images are shown, see Supplementary Fig. 16 for full immunoblots). **I** Representative gait test images measured in WT and STING^gt/gt^ mice; *n* = 9 biologically independent animals. **J** Representat**i**ve immunoblots and quantification of TH, DJ1, Nrf2 in SNc of WT and STING^gt/gt^ mice (cropped blot images are shown, see Supplementary Fig. 16 for full immunoblots). Data are mean ± s.e.m.; *n* = 9 biologically independent animals; *P < 0.05, **P < 0.01, and ***P < 0.001 by two-way ANOVAs followed by Tukey’s multiple comparisons test. **K** Relative mRNA expression in SNc of WT and STING^gt/gt^ mice. Data are mean ± s.e.m.; *n* = 6 biologically independent animals. **L** Time to traverse beam apparatus, time to descend pole, hind-limb clasping reflex score, fall latency from an accelerating rotarod. Data are mean ± s.e.m.; *n* = 10 biologically independent animals; *P < 0.05, **P < 0.01, and ***P < 0.001 by two-way ANOVAs followed by Tukey’s multiple comparisons test. **M** Schematic summary of the mechanism underlying the neuroprotective actions of WA in PD.

To determine whether STING underpins the effects of WA, we overexpressed STING in SNc by bilateral intra-SNc injection of DMXAA, a murine STING agonist [36]. DMXAA administration led to a high level of STING, along with an activation of microglia (Fig. 5C, D). In MPTP-treated mice, DMXAA caused a profound loss of dopaminergic neurons (36% versus 52%) (Fig. 5A, B), a reduction in TH protein levels (Fig. 5H), and an impaired motor function (Fig. 5E). The mice received intra-SNc injection of DMXAA alone without MPTP appeared no obvious PD-like symptoms (Fig. 5A). In STING^gt/gt^ mice and STING-KO mice, the loss of dopaminergic neurons (64% versus 50% in STING^gt/gt^ mice, and 66% versus 49% in STING-KO mice) (Fig. 5F, G, Supplementary Fig. 9A), the reduction in density of TH^+^ fibers (Supplementary Figs. 8 and 9) were attenuated. The decreased TH and DJ1 expression levels (Fig. 5J) and the impaired motor function (Fig. 5L and Supplementary Fig. 9B) were mitigated in STING^gt/gt^ or STING^−/−^ mice treated with MPTP. The effects of WA on astrocytes and microglia were dampened by DMXAA (Fig. 5C, D). The activation of astrocytes and microglia induced by MPTP was relieved in SNc and STR (Fig. 5F, G, Supplementary Fig. 8), and the high levels of inflammatory factors were ameliorated in SNc of STING^gt/gt^ mice (Fig. 5K). WA led to a small but insignificantly higher anti-inflammatory effect in STING^gt/gt^ mice treated with MPTP (Fig. 5F, G, K). WA had little effect on the high level of STING induced by MPTP in both DJ1-KO mice and Nrf2-KO mice (Figs. 3 and 4). WA induced marked expressions of DJ1 and Nrf2 in SNc of STING^gt/gt^ mice received MPTP (Fig. 5J, K). In human dopaminergic neurons, STING knockdown had no obvious effect on the activation of DJ1 and Nrf2 induced by WA in response to MPP^+^ (Supplementary Fig. 12). WA caused a decrease in STING in dopaminergic neurons of MPTP-treated mice (Supplementary Figs. 10A and 11B), whereas WA had slight effect on heavy STING immunoreactivity in microglia (Supplementary Fig. 11A, B), and did not affect the immunoreactivity of STING in astrocytes (Supplementary Fig. 10B). Similarly, WA normalized the STING immunoreactivity to control levels in MPP^+^ treated human dopaminergic neurons (Supplementary Fig. 12). The absence of STING resulted in a substantial decrease in inflammatory, STING-related and apoptotic genes in SNc of MPTP-treated mice; whereas, the mitigative effects of WA on these genes was unchanged (Fig. 5K). The absence of STING had no overt effect on the anti-oxidative genes suppressed by MPTP, and this suppression could be mitigated by WA (Fig. 5K). These results demonstrate that STING, as a novel downstream factor of DJ1-Nrf2 pathway, mediates the anti-neuroinflammatory actions of WA, revealing that STING may be a novel therapeutic target to mitigate PD-like symptoms.

### WA prevents degeneration of dopaminergic neurons overexpressing α-syn

To model the time course of the progression from early toward advanced stages of PD patients [37, 38], we performed AAV-mediated human wild-type α-syn (h-α-syn) overexpression within SNc, as shown in Supplementary Fig. 13A, D. As expected, co-labeling with TH (red) and h-α-syn (blue) confirmed that h-α-syn was expressed in the majority of dopaminergic neurons at 21 days post injection (Supplementary Fig. 13C). As previously described [37], h-α-syn overexpression induced a loss of TH^+^ neurons (~52% loss) in SNc and a reduced density of TH^+^ fibers in STR (Supplementary Fig. 13B). As previously reported, α-syn was also observed in axon terminals in striatum suggesting that proteins produced in the nigral dopaminergic neurons were transported anterogradely towards the synaptic terminals (Supplementary Fig. 15) [39, 40]. The loss of TH^+^ neurons (Supplementary Fig. 13E), the reduction in TH and DAT immunoreactivity (Supplementary Fig. 13I, J), and the impaired motor function (Supplementary Fig. 13N) in response to h-α-syn overexpression were relieved by WA. WA reduced the amount of pathologic Triton X-100 insoluble α-syn and p-α-syn in AAV-h-α-syn received mice (Supplementary Fig. 13K, L). WA decreased p-α-syn immunoreactivity in SNc, specifically in TH immunoreactive neurons. (Supplementary Fig. 13G, H and Supplementary Fig. 14). The h-α-syn overexpression decreased the levels of genes associated with ALP and UPS, which was restored by WA (Supplementary Fig. 13M). The high levels of genes related to neuroinflammation and apoptosis, and the low levels of genes associated with mitochondrial function triggered by h-α-syn overexpression were relieved by WA (Supplementary Fig. 13M). These results suggest that WA may alleviate α-syn pathology to impede the dopaminergic neuronal degeneration by enhancing ALP and UPS function, highlight that WA may represent a multi-effective therapeutic agent to alleviate progressive dopaminergic degeneration in PD.

### WA and amantadine may treat PD by suppressing STING

**Fig. 6:**
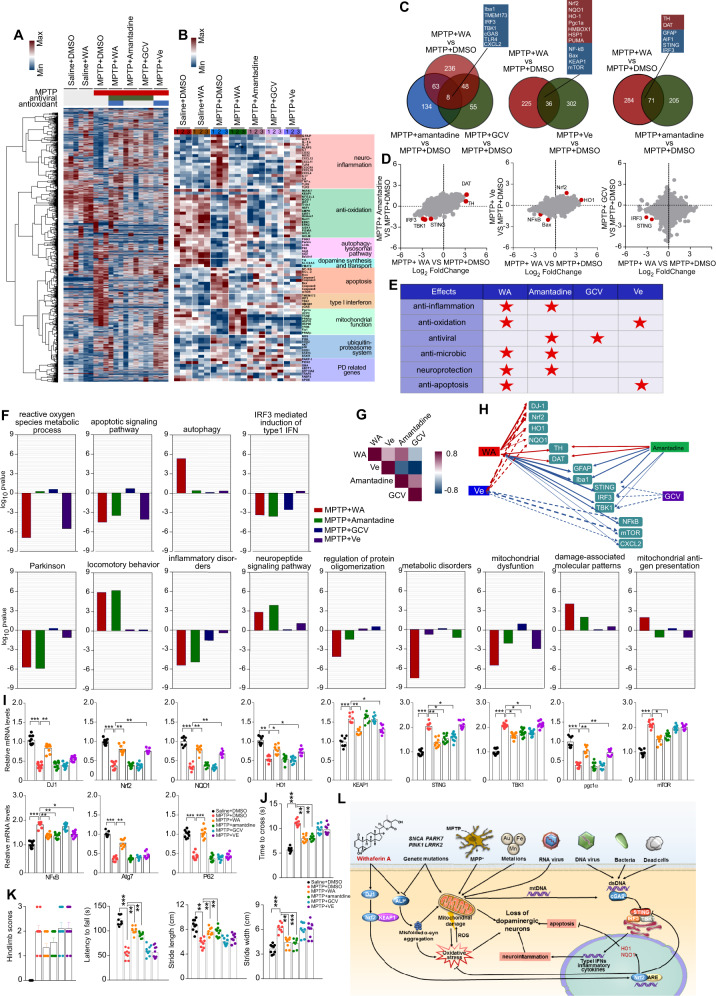
Withaferin A has multiple neuroprotective effects; Withaferin A and amantadine may treat PD by suppressing STING. **A** Hierarchical clustered heatmap of gene expression profiles for WA, amantadine, Ve, ganciclovir treatment in SNc of MPTP-induced PD mice. **B** The heatmap of DEGs of MPTP-received mice treated with WA, amantadine, Ve, ganciclovir. **C** Venn diagram of overlapping significantly changed genes (±1.2 fold, *P* < 0.05). **D** Scatter plot highlights the DEGs of amantadine, Ve and GCV treatment compared with vehicle in MPTP-recieved mice, significantly altered genes are colored in red. (*P* < 0.05 with Benjamini–Hochberg multiple testing correction). **E** The pharmacological actions of WA, amantadine, Ve and GCV from published literatures. **F** Gene Ontology enrichment was based on DEGs that have a *P* value smaller than 0.05. **G** Spearman correlation between aggregated response of WA, amantadine, Ve and GCV gene signatures to different interventions. **H** Network of interventions based on similarity of their gene expression profiles. The width of edge is defined by statistical significance of Spearman correlation between interventions. Only significant connections are shown. **I** Relative mRNA levels in SNc of WA, amantadine, Ve and GCV treated mice. Data are mean ± s.e.m.; *n* = 9 biologically independent animals; *P < 0.05, **P < 0.01, and ***P < 0.001 by one-way ANOVA with Bonferroni’s post hoc test. **J**, **K** Time to traverse beam apparatus, time to descend pole, hind-limb clasping reflex score, fall latency from an accelerating rotarod and gait analysis. Data are mean ± s.e.m.; *n* = 9 biologically independent animals. The one-way ANOVAs were used for statistical analysis followed by Bonferroni’s post hoc test. *P < 0.05, **P < 0.01, and ***P < 0.001. **L** Schematic summary of the mechanism underlying the neuroprotective actions of WA in PD. The neuroprotective role of WA against PD is dependent on relief of oxidative stress, mitigation of the STING-mediated neuroinflammation, enhancement of mitochondrial function, and reduction of apoptosis, through activation of DJ1-Nrf2-STING axis in dopaminergic neurons of SNc.

Amantadine is first approved as an antiviral agent, which is also used in PD treatment, but the mechanism underlies its neuroprotective effect against PD remains elusive [31]. We found that the motor impairment was mitigated by WA or amantadine (Fig. 6J, K). Vitamin-E (VE, an antioxidant control drug) and ganciclovir (GCV, an antiviral control drug) modestly attenuated motor deficit in MPTP-induced PD mouse model (Fig. 6J, K). The transcriptome sequencing analysis showed that each agent exhibited its own expression pattern of responsive genes (Fig. 6A, B). Unsupervised hierarchical clustering revealed that the alterations of STING-dependent response genes, the neuroinflammatory genes, and the genes associated with dopamine synthesis were restored by WA or amantadine. The STING-dependent response genes and the neuroinflammatory genes were also downregulated by GCV. VE treatment led to a relief of the genes related to oxidation and apoptosis (Fig. 6A, B). Venn diagram and Scatter plot show that these agents shared many DEGs. WA and amantadine elevated expressions of the genes associated with dopamine synthesis (Fig. 6C, D). WA, amantadine and GCV led to a suppression of STING-dependent response genes. WA and VE normalized the genes linked to oxidative stress and apoptosis (Fig. 6C, D). The alterations of genes expression were confirmed by qPCR, as shown in Fig. 6I. GO and KEGG analyses show that WA, amantadine and GCV inhibited the pathways relative to STING-dependent neuroinflammation; WA and amantadine relieved apoptosis, enhanced neuropeptide signal and dopamine synthesis pathways (Fig. 6F). WA and VE normalized anti-oxidative, mitochondrial, and apoptotic pathways. Autophagy-related genes and mitochondrial antigen presentation-related pathway were enhanced by WA (Fig. 6F). The pharmacological actions of these agents were summarized in Fig. 6E. The high degree of correlation of aggregated gene expression profiles across agents showed that WA shares partial pharmacological actions with amantadine and VE (Fig. 6G, H). As the diagram illustrates in Fig. 6L, our findings suggest that the neuroprotective role of WA against PD is dependent on the relief of oxidative stress, the mitigation of STING-mediated neuroinflammation, the enhancement of mitochondrial function, and the reduction in apoptosis, which is mainly through DJ1-Nrf2-STING axis in dopaminergic neurons of SNc. In addition, WA decreases the aggregation of p-α-syn and insoluble α-syn, enhances the ALP and UPS function. Further, the activation of STING, which occurs in response to damaged DNA, exacerbates PD pathogenesis, while WA and amantadine may suppress STING to protect against PD.

## Discussion

The major findings of this paper are the observation that WA protects against PD. Recently, both the dopaminergic and neuroinflammation-targeting therapies have emerged to play key roles in the development of PD treatment [41–43]. Our findings show that WA treatment leads to a restoration of the genes associated with dopamine synthesis and transport, and also a relief of the genes related to neuroinflammation in SNc, which may contribute to the neuroprotective effects of WA against PD.

Importantly, we found that WA may activate DJ1-Nrf2 axis and suppress STING in SNc dopaminergic neurons; and Meta-analysis of genes expression in SNc also revealed that the level of STING surges in SNc of PD patients. These suggest that STING is an important therapeutic target, and also support that DJ1, Nrf2 and STING are associated with PD pathogenesis.

The DJ1 and Nrf2 participate in anti-oxidative, anti-inflammatory and anti-apoptotic processes under the condition of PD-causing toxins [44]. DJ1 stabilizes Nrf2 by preventing its ubiquitination, and promotes the expressions of Nrf2-dependent anti-oxidative and anti-inflammatory genes, such as *HO1, NQO1* [45, 46]. We found that WA normalizes the decreased DJ1 and Nrf2 levels in SNc and protects against the loss of dopaminergic neurons and the neuroinflammation, indicating that DJ1-Nrf2 axis mediates the neuroprotective actions of WA.

In this study, we found that WA suppresses the STING expression triggered by MPTP; whereas activation of STING exacerbates the PD-like symptoms and impairs the neuroprotective actions of WA. STING deficiency relieves the loss of dopaminergic neurons in PD mice. Emerging evidence suggests that STING may act as a critical factor in neuroimmunomodulation [24]. The neuroinflammation mediated by STING exacerbates the pathogenesis of Huntington Disease [47]. STING is also activated by DAMPs or PAMPs, such as viral DNA, or leaked mitochondrial DNA. It is a fact that there are a variety of viruses which have the ability to infect CNS, and some of the viruses are retained in neurons for long periods, or even a lifetime, such as herpes simplex-1, varicella-zoster virus, sindbis virus, measles and rabies [3, 48, 49]. The vulnerability of CNS to infectious agents increases with aging [3]. Even transient viral infection triggers neurodegenerative cascades when the infection occurs in aged individuals [3]. Strikingly, the antiviral therapy decreases the incidence of PD and delays the onset of PD in HCV (Hepatitis C Virus) infected patients [32]. PD, HCV infection and neuroinflammation mediated by STING share similar biomarkers, such as IL6, IL8 and TNFα in serum [24, 32]. Antiviral agents suppress the STING expression [50]. We show that WA relieves the STING level in SNc of PD mice, and this is also verified in human dopaminergic neurons. Amantadine, an antiviral drug, is also an anti-PD agent. The mechanism underlying neuroprotective actions of amantadine remains elusive [7, 31]. We found that WA and amantadine suppress the STING-mediated neuroinflammation; and WA and amantadine share a considerable amount of transcriptomic profiles in SNc. WA and amantadine treatments lead to elevation of the genes associated with dopamine synthesis; WA, amantadine and GCV cause suppression of the STING-dependent response genes in PD mice. WA and Ve mitigate the levels of anti-oxidative genes, the apoptotic genes, and restore the mitochondrial genes. Collectively, we propose that STING may be a novel factor involved in PD pathogenesis; the viral infection may be a novel risk factor for PD; and STING may be linked to the neuroprotective effects of WA or amantadine. We suggest a potential therapeutic application of antiviral agents for neurodegenerative diseases, especially for PD. These findings suggest a novel mechanism underlying effects of WA and amantadine against PD, and highlight the importance of STING-mediated neuroinflammation in PD pathogenesis.

Recent studies revealed that Nrf2 negatively regulates STING by decreasing STING mRNA stability, and Nrf2 activators repress STING-mediated type I IFN production [51]. Nrf2 activator mitigates inflammation by inhibiting STING-dependent NFκB signaling [52]. In this study, we show that WA inhibits the STING-mediated neuroinflammation through DJ1-Nrf2 pathway; and WA activates DJ1-Nrf2-STING axis predominately in dopaminergic neurons.

To model the degenerative feature of PD and to ascertain the neuroprotective action of WA in PD, we performed AAV vectors-mediated human wild-type α-syn overexpression within the SNc; owing to the accumulation of α-synuclein and protein degradation defects are pathological hallmark of PD [5, 6]. Although neurotoxic PD models are useful for preclinical validation of new therapeutic agents; they fail to recapitulate the spread of α-synuclein-mediated pathology that occurs in PD human patients [5]. Our findings demonstrate that WA decreases the pathological insoluble α-syn and ser129 p-α-syn, and enhances the gene expressions associated with ALP and UPS system in SNc of human α-syn overexpression PD model. Moreover, WA relieves the impaired nigrostriatal and motor function. These data suggest that promotion of ALP and UPS is  implicated in anti-PD effects of WA.

In summary, this work identified that WA exerts anti-PD effects through DJ1, Nrf2, and STING pathways. STING may be a potential target of WA and amantadine. STING-mediated neuroinflammation may play a critical role in PD pathogenesis. Our study highlights a broad neuroprotective action of WA via multiple mechanisms against PD.

## Methods

### Mice

C57BL/6 mice were purchased from Charles River Laboratories Beijing Branch (Beijing Vital River Laboratory Animal Technology Co., Ltd.) and the Department of Laboratory Animal Science of Peking University Health Science Center. Male mice were used for all the experiments at 8–10 weeks of age described in this study. We maintained mice on a 12 h light-dark cycle in a temperature-controlled high barrier facility with unrestricted access to food and water. DJ-1 knockout mice with a C57BL/6/CBA background were a kind gift from Dr Zengqiang Yuan of Chinese Academy of Sciences [53]. Nrf2-KO knockout mice with a C57BL/6/CBA background were kindly provided by Dr Siwang Yu of Peking University [54]. STING^gt/gt^ mice were a kind gift from Dr Fuping You of Peking University [55]. STING knockout mice were a kind gift from Dr Zhengfan Jiang of Peking University [56]. All mouse work in this study was performed under the guidelines of the Ethics Committee of Peking University Health Science Center (PKUHSC) (LA2016113). All animal care and use followed the guidelines of the Animal Care and Use Committee of Peking University. During all procedures of experiments, the number of animals and their suffering by treatments were minimized.

### Agent treatments

Eight to ten weeks old male mice weighing 24–28 g were housed under standard conditions. After 1 week acclimatization, mice were injected with MPTP at a dose of 30 mg/kg i.p. for 5 days, as used previously [57], and the controls were administered an equal volume of saline (0.9% NaCl). Then mice were administered Withaferin A (ChromaDex Irvine, CA) at dose of 2, 20, 200, 2000 μg/kg for 7, 14, 21 days. Withaferin A was dissolved in DMSO (25 µl) and administered to the mice intraperitoneally once a day, as previously reported [58]. Amantadine (Energy chemical, A109736) was dissolved in saline, and mice were administered at dose of 25 mg/kg for 7 days post MPTP injection, the chosen dosage was previously reported [59]. Ganciclovir (Targetmol, T0688) was dissolved in PBS, and 100 mg/kg of the solution was intraperitoneally injected for 7 days [60]. Vitamin E (Solarbio, V8010-5) was dissolved in DMSO, and 100 mg/kg of the solution was intraperitoneally injected for 7 days post MPTP administration.

### Cell culture, transfection, and treatment

Cells were maintained in Dulbecco’s Modified Eagle’s Medium/Nutrient Mixture F-12 (DMEM/F12; Hyclone, SH30023.01B), to which 10% fetal bovine serum (PAN-Biotech, P30-2602) and antibiotics (100 U/ml penicillin, 100 μg/ml streptomycin; Thermo Fisher Scientific, 15140122) had been added. Cells were grown at 37 °C under an atmosphere of 5% CO_2_. Gene knockdown achieved by using siRNAs directed against human DJ1, Nrf2, and STING were synthesized by Hanbio, the sequences of the siRNA were as follows: siDJ-1 (5’ -UGGAGACGGUCAUCCCUGUdTdT-3’) [61], siNrf2 (5’-GUAAGAAGCCAGAUGUUAAdUdU-3’) [62] and siSTING (5’-GCAACAGCAUCUAUGAGCUUCUGGAGAAC-3’) [63]. siRNAs and FAM negative control siRNA (Hanbio) were transiently transfected into SH-SY5Y cells by using Lipofectamine 2000 (Invitrogen, 11668019) according to the manufacturer’s protocols. Concentrations of siRNAs were chosen on the basis of dose–response studies (data not shown).

### Cell death and viability assessment

Cell viability was tested using two methods. (1) Propidium iodide staining. Tansfected SH-SY5Y cells were treated with 1 mm MPP^+^ (Sigma, D048) or MPP^+^ with 2 μm WA for 24 h. Cell viability was determined by unbiased objective computer-assisted cell counting after staining of all nuclei with 7 μM Hoechst 33342 (Invitrogen) and dead cell nuclei with 2 μm propidium iodide (Solarbio, C0080-10) [64]. Image J software was used to analyze the data, the percentage of cell death was determined as the ratio of live to dead cells compared to the percentage of cell death in control wells to account for cell death attributed to MPP^+^ neurotoxicity. (2) MTT assay. The cell viability was also quantified by a 3-(4,5-dimethylthiazol-2-yl)-2,5-diphenyltetrazolium bromide (MTT, Beyotime, ST316) assay. Briefly, cells were plated at a density of 1 × 10^4^ cells per well into 96-well plates and maintained at 37 °C for 24 h, then cells were treated with 1 mm MPP^+^ (Sigma, D048) or MPP^+^ with 2 μm WA for 24 h, followed by the further incubation with 0.5% MTT solution (5 mg/ml) for 4 h. After the medium was removed and regular medium was added to prevent the drugs from reacting directly with MTT, the cells and formazan were dissolved by adding dimethylsulfoxide (DMSO), and the light absorbance was measured at 490 nm in a microtiter plate reader.

### Immunohistochemistry and immunofluorescence

Immunohistochemistry and immunofluorescence were performed on 30-μm thick serial brain sections. Primary antibodies and working dilutions are detailed in Table S1. Mice were perfused with PBS and 4% PFA and brains were removed, followed by fixation in 4% PFA overnight and transfer to 20–30% gradient sucrose for cryoprotection, and subsequently freezed in OCT compound (Sakura FineTech, Tokyo). For histological studies, brain slices were blocked with 10% goat serum in PBS with 0.2% Triton X-100 and incubated with TH, GFAP or Iba1 antibodies. After washing with PBS three times, brain tissues were used appropriate biotin secondary antibody, followed by avidin-biotin complex (Zsbio, SP-9001) and visualized with 3,3’-diaminobenzidine (DAB) peroxidase substrate (Zsbio, ZLI-9018). Sections were counterstained with Nissl staining solution (Beyotime, C0117), and photographed by light microscope (Leica DMI 4000B, Wetzlar, Germany). The number of TH- and Nissl-positive dopaminergic neurons, and the number of microglia and astrocytes in the SNpc region, and the density of TH positive fibers in the STR were measured with ImageJ software. For immunofluorescent studies, dual-antigen immunofluorescence was performed to detect the expression level of DJ1, Nrf2 or STING in dopaminergic neurons, astrocytes or microglia, using following antibodies: mouse anti-DJ1 (1:200, Santa Cruz), rabbit anti-Nrf2 (1:500, Abcam), mouse anti-Nrf2 (1:200, Santa Cruz), rabbit anti-STING (1:500, Proteintech), mouse anti-STING (1:500, Proteintech), rabbit anti-TH (1:500, Millipore), mouse anti-TH (1:500, Santa Cruz), rabbit anti-GFAP (1:500, Bioss), mouse anti-GFAP (1:200, Snata Cruz), rabbit anti-Iba1 (1:500, Wako), mouse anti-Iba1 (1:500, GeneTex). Sections were washed in PBS and incubated with a mixture of Alexa-fluor 488- and 594- conjugated secondary antibodies (1:500, YEASEN) at room temperature for 120 min. Sections were mounted with VectaShield medium (Vector Laboratories) and analyzed by detecting fluorescein using a fluorescence microscope (Leica DMI 4000B, Wetzlar, Germany).The selected area in the signal intensity range of the threshold was measured using ImageJ software.

### Total protein extraction and western analysis

SNc and STR were homogenized with a Polytron in ice-cold RIPA buffer [1% Trinton X 100; 10 mM Na2HPO4 (Sodium phosphate); 150 mM NaCl (Sodium chloride); 1% DOC (Sodium deoxycholate or deoxycholic acid); 5 mM EDTA; 5 mM NaF (sodium Fluoride); 0.1% SDS] supplemented with protease and phosphatase inhibitors (catalog #P8340 and #P2850; Sigma), sonicated and cleared by centrifugation (10,000 × *g*, 10 min, at 4 °C). Protein concentration in the supernatant was determined by BCA assay (Aidlab; PP01). Protein (5 μg) in 1 × sample buffer [62.5 mM Tris•Cl (pH 6.8), 2% (wt/vol) SDS, 5% glycerol, 0.05% (wt/vol) bromophenol blue] was denatured by boiling at 100 °C for 5 min and separated on 8% sodium dodecyl sulfate poly acrylamide (SDS-PAGE) gels and transferred onto nitrocellulose membrane (Pall Corporation; T60327) by electrophoresis. Blots were blocked in 5% nonfat milk in Tris-buffered saline and Tween 20 (TBST) for 2 h at room temperature and probed with primary antibody in 5% BSA-TSBT overnight at 4 °C. After overnight incubation, the blots were washed three times in TBST for 15 min, followed by incubation with HRP-conjugated secondary antibody in TBST with 5% nonfat milk for 2 h at room temperature. Following three cycles of 15 min washes with TBST, the blots were developed using an Enhanced Chemiluminescence assay (BIO-Rad). Densitometry analysis was performed on scanned western blot images using the ImageJ software (NIH). All raw blot and gel images are available in Supplementary Figs. 16 and 17.

### α-synuclein protein sequential extraction protocol

Dissected SNc regions were prepared with sequential lysis buffers. For the soluble fraction, samples were homogenized in the following TX-soluble buffer (50 mM Tris [pH 8.0], 150 mM NaCl, 1% Triton-100) containing protease and phosphatase inhibitors (catalog #P8340 and #P2850; Sigma) and samples were centrifuged and the soluble supernatant was collected. The insoluble pellet was resuspended in TX-insoluble buffer (50 mM Tris [pH 8.0], 150 mM NaCl, 1% Triton X-100, 2% SDS) containing protease and phosphatase inhibitors (Roche, New York, NY, USA). Samples were sonicated and centrifuged at 20,000 × *g* for 20 min. Protein concentrations were determined using the BCA assay and protein (10 μg) were separated on SDS-polyacrylamide gels and transferred onto nitrocellulose membranes. Blots were blocked in 5% nonfat milk in Tris-buffered saline and Tween 20 (TBST) for 2 h at room temperature and probed with primary antibody in 5% BSA-TSBT overnight at 4 °C. After overnight incubation, the blots were washed three times in TBST for 15 min, followed by incubation with HRP-conjugated secondary antibody in TBST with 5% nonfat milk for 2 h at room temperature. Following three cycles of 15 min washes with TBST, the blots were developed using an Enhanced Chemiluminescence assay (BIO-Rad). Densitometry analysis was performed on scanned western blot images using the ImageJ software.

### Behavioral tests

To evaluate the beneficial effect of WA on behavioral deficits in MPTP-induced PD mouse model and AAV vectors-mediated human α-syn overexpression induced PD model, mice were assessed by beam traversal, pole test, rotarod test, hindlimb scoring and gait test. Motor function for all animals was tested between 10:00–16:00 in the lights-on cycle. All tests were performed similarly to previous studies [64, 65].

### Beam traversal

A 1 m beam was constructed of four segments of 0.25 m in length. Each segment was of thinner widths 3.5 cm, 2.5 cm, 1.5 cm, and 0.5 cm, with 1 cm overhangs placed 1 cm below the surface of the beam. The widest segment acted as a loading platform for the animals and the narrowest end placed into home cage. Animals had two days of training to traverse the length of the beam before testing. On the first day of training, animals received 1 trial with the home cage positioned close to the loading platform and guided the animals forward along the narrowing beam. Animals received two more trials with limited or no assistance to encourage forward movement and stability on the beam. On the second day of training, animals had three trials to traverse the beam and generally did not require assistance in forward movement. On the third day, animals were timed over three trials to traverse from the loading platform and to the home cage. Timing began when the animals placed their forelimbs onto the 2.5 cm segment and ended when one forelimb reached the home cage.

### Pole test

Mice were acclimatized in the behavioral procedure room for at least 30 min. The pole a 75-cm metal rod with a diameter of 9 mm. It was wrapped with bandage gauze. Mice were placed near the top of the pole (7.5 cm from the top of the pole) facing upwards. The total time taken to reach the base of the pole was recorded. Before the actual test, mice were trained for two consecutive days. Each training session consisted of three test trials. On the test day, mice were evaluated in three sessions and the total time was recorded. The maximum cutoff time to stop the test and recording was 60 s. Results for turn down, climb down and total time (s) were recorded.

### Rotarod test

For the rotarod test, mice were placed on an accelerating rotarod cylinder, and the time the animals remained on the rotarod was measured. The speed was slowly increased from 4 to 40 r.p.m. within 5 min. A trial ended if the animal fell off the rungs or gripped the device and spun around for two consecutive revolutions without attempting to walk on the rungs. The animals were trained 3 days before the test. Motor test data are presented as the percentage of the mean duration (three trials) on the rotarod compared to the control.

### Hindlimb scoring

Animals were gently lifted upward by the mid-section of the tail and observed over 5–10 s. Animals were assigned a score of 0, 1, 2, 3 based on the extent to which the hindlimbs clasped inward. 0, indicating no clasping, was given to animals that freely moved both their limbs and extended them outward. A score of 1 was assigned to animals which clasped one hindlimb inward for the duration of the restraint or if both legs exhibited partial inward clasping. A score of 2 was given if both legs clasped inward for the majority of the observation, but still exhibited some flexibility. A score of 3 was assigned if animals displayed complete paralysis of hindlimbs that immediately clasped inward and exhibited no signs of flexibility.

### Gait test

The testing apparatus is made of a gray acrylic board (3 mm thick), and consists of a runway (10 cm width, 60 cm length, 12 cm height) with non-slippery white paper and a dark goal box (16 cm width, 10 cm length, 12 cm height). On the first training day, mice were habituated to the apparatus for 2 min, then their forepaws and hindpaws were painted red and black with non-toxic food dyes and trained to run to the goal box (training trial). A training trial was performed once a day for two consecutive days before MPTP administration. In test trials, mice were made to run the runway in the same manner (cut-off time 60 s maximum). The footprint patterns were analyzed for three parameters (stride length, stride width, and overlap), prints near the start and the goal being excluded because of the effects of acceleration or deceleration. Stride length was measured as the average distance between each forepaw and hindpaw footprint. Stride width was measured as the average distance between the right and left footprint of each forepaw and hindpaw. Overlap was measured as the average distance between the center of forepaw and hindpaw footprints on the same side. At least four values were measured in each trial for each parameter.

### Dopamine and derivatives measurement using HPLC

High-performance liquid chromatography with electrochemical detection (HPLC-ECD) was used to measure dopamine, 3,4-dihydroxyphenyl acetic acid (DOPAC), and homovanillic acid (HVA) concentrations. Briefly, the striatum was rapidly removed from the brain, followed by weighing, then sonication in ice cold 0.01 mM of perchloric acid containing 0.01% EDTA. This was followed by centrifugation (20,000 × *g*, 20 min, 4 °C) and passing the supernatant through a 0.2 μm filter. The mobile phase consisted of 85 mM citric acid, 100 mM anhydrous acetic sodium, 0.2 mM ethylenediamine teraacetic acid disodium salt (EDTA-2Na) and 15% (v/v) methanol (pH 3.68) at a flow rate of 1.2 mL/min. Concentrations of DA and its metabolites were expressed as μg/g tissue weight.

### Quantitative real-time PCR

Total RNA was extracted from adipose tissues and hypothalamus using TRIzol reagent (TransGen Biotech). Quantification and integrity analysis of total RNA was performed by running 1 µl of each sample on NanoDrop 5500 (Thermo). The cDNA was prepared by reverse transcription (TransScript one-step gDNA removel and cDNA synthesis Super MiX, TransGen Biotech). The relative expression of mRNAs was determined by the SYBR Green PCR system (Bio-Rad). The relative expression of genes of interest was calculated by comparative Ct method and GAPDH was used as an endogenous control. GAPDH RNA was chosen as the housekeeping gene. Sequences of the primers used for real-time qPCR are available in Table S2: Primers used in the present study.

### Whole-genome sequencing analysis

Total RNA was extracted from SNc using TRIzol reagent (TransGen Biotech). Quantification and integrity analysis of total RNA was performed by running 1 µl of each sample on NanoDrop 5500 (Thermo). A total amount of 3 μg RNA per sample was used as input material for the RNA sample preparations. After that, the RNAs were subjected to 50-bp single-end sequencing with a BGISEQ-500 sequencer as previously described [66]. At least 20 million clean reads of sequencing depth were obtained for each sample. Differential expression analysis of two groups (two biological replicates per condition) was performed using the DESeq R package (1.10.1). DESeq provide statistical routines for determining differential expression in digital gene expression data using a model based on the negative binomial distribution. The resulting *P* values were adjusted using the Benjamini and Hochberg’s approach for controlling the false discovery rate. Genes with an adjusted *P* value < 0.05 found by DESeq were assigned as differentially expressed. DEGs were defined as genes with FDR less than 0.01 and log2 fold change larger than 1 (upregulation) or smaller than -1 (downregulation). Gene Ontology (GO) and pathway annotation and enrichment analyses were based on the NCBI COG (https://www.ncbi.nlm.nih.gov/COG/), Gene Ontology Database (http://www.geneontology.org/) and KEGG pathway database (http://www.genome.jp/kegg/), respectively. The software Cluster and Java Treeview were used for hierarchical cluster analysis of gene expression patterns.

### Meta-analysis

A total of twelve striatal transcriptomic datasets were identified (GSE42966, GSE49036, GSE43490, GSE28894, GSE20333, GSE20141, GSE7621, GSE41569228, GSE54282, GSE8397, GSE77666, GSE77668). Log2-transformation was applied as needed. In order to control for the effects of known and hidden covariates in each of the datasets, we used R/sva package to first adjust expression data for known factor covariates (such as sex) with the combat function and then estimate surrogates for hidden covariates with the sva function. Known numeric covariates (such as age and postmortem interval, when available) and estimated surrogates were then evaluated by fitting the expression data a linear model and comparing the distribution of model P values against uniform distribution, in order to select covariates with widespread effects. Selected covariates were further adjusted for by fitting the expression data with a robust linear model and taking intercept + residuals as the adjusted expression values. Within each dataset, the expression values were further standardized to μ = 0 and σ = 1, and effect sizes (Hedges’ g) of disease status were then computed for each gene. We condensed the expression profile to the gene level, keeping the probeset with the largest effect size (i.e., the smallest dataset-specific *P* value and thus the least likely by chance) to represent the gene. We used the R/GeneMeta package to combine effect sizes across datasets with a random effect model, compute meta-Z-score statistics, and estimate false discovery rate (FDR) by 1000 permutations.

### Adeno-associated virus-induced overexpression of human alpha-synuclein

Recombinant AAVs (serotype 2 genome packaged in serotype 9 capsid) were used for the expression of human wild-type α-syn, GFP driven by the human synapsin-1 promoter and enhanced using a woodchuck hepatitis virus posttranscriptional regulatory element (WPRE). Injected vector titre was 1 × 10^14^ genome copies/ml(gc/ml) for both AAV-h-α-syn and AAV-GFP vectors [37].

### Stereotaxic injection

Vector solution was bilaterally injected within the SNc region using a 0.2 mm-gauge stainless steel injector connected to a 5 μl Hamilton syringe. In all experimental groups, the AAV was injected in a volume of 1 μl/side at a rate of 0.2 μl/min. The stereotaxic coordinates used (flat skull position) were: AP = −3.2 mm; ML = ±1.2 mm, DV = −4.6 mm relative to the bregma, according to the atlas of Paxinos and Franklin (2001). Only animals with correct injection placements, verified by analyzing immunofluorescence staining of consecutive coronal brain sections, were included in the statistical analysis transgene expression of human wildtype alpha-synuclein. AAV production and titration were performed by OBiO Technology.

### Cannula placement and DMXAA administration

DMXAA (5, 6-dimethylxanthenone-4-acetic acid, Vadimezan, T6273, Targetmol) was dissolved in 50 mM Tris buffer at a concentration of 10 mg/ml and the pH was adjusted to 7.8 – 8.2. For the used concentrations DMXAA stock solution was diluted with PBS. After acclimation, mice were anesthetized with isoflurane and were implanted with guide cannulas in the SNc (AP = −3.2 mm; ML = ±1.2 mm, DV = −4.6 mm) according to Paxinos and Watson’s (2001) stereotaxic atlas. The cannula was fixed in place with dental cement and secured by two skull screws and small round-top dust caps. Mice were allowed a recovery period of 7 days before the experiments. All injections were performed in freely moving animals at a rate of 0.25 μl/min using a dual syringe. Following infusion, the cannula was left in place for an additional 1 min to minimize dragging of infused liquid along the infusion track. The infusion cannula was 0.5 mm longer than the guide cannula.

### Statistical analysis

Data are expressed as the mean ± s.e.m. with at least three biologically independent experiments. Representative morphological images were taken from at least three biologically independent experiments with similar results. Statistical significance was determined using an unpaired two-tailed Student *t* test, one-way or two-way ANOVA, and then either Tukey’s or Bonferroni’s multiple comparison test was performed to compare all treatment groups. All data were analyzed using the appropriate statistical analysis methods, as specified in the figure legends, with the SPSS software (version 19.0). Significance was accepted at * *P* < 0.05, ** *P* < 0.01 or *** *P* < 0.001.

## Supplementary information

Supplementary file

Supplementary Fig 1

Supplementary Fig 2

Supplementary Fig 3

Supplementary Fig 4

Supplementary Fig 5

Supplementary Fig 6

Supplementary Fig 7

Supplementary Fig 8

Supplementary Fig 9

Supplementary Fig 10

Supplementary Fig 11

Supplementary Fig 12

Supplementary Fig 13

Supplementary Fig 14

Supplementary Fig 15

Supplementary Fig 16

Supplementary Fig 17

Supplementary Tables
