# Supplementary material for: The DJ1-Nrf2-STING axis mediates the neuroprotective effects of Withaferin A in Parkinson’s disease
Source: Cell Death Differ. 2021 Mar 24;28(8):2517–35. doi: 10.1038/s41418-021-00767-2 (PMC8329302; doi:10.1038/s41418-021-00767-2)
Supplement: Supplementary file 1 — Supplementary file [file 41418_2021_767_MOESM1_ESM.doc]

**Supplementary Materials for**

**The DJ1-Nrf2-STING axis mediates the neuroprotective effects of Withaferin A in Parkinson's disease**

**Legends for supplementary Fig. 1 - 17
Table S1 – 2 (included in the article file)**

**Supplementary Fig. 1 Neuroprotective role of Withaferin A is dose-dependent.** (**A**) Diagram of the experimental design. MPTP (20 mg/kg）or vehicle (saline) was injected (i.p.) for 5 consecutive days starting on day -4 after acclimation (3 days), then mice intraperitoneally (i.p.) received Withaferin A (WA) (2, 20, 200, 2000 μg/kg) or vehicle (DMSO) per day for 7 days, tissues were harvested for molecular analyses at day 8 after the last behavior test. (**B**) Representative immunoblots of TH and DAT and β-actin and quantification of TH and DAT protein levels at the doses of 2, 20, 200 and 2000 μg/kg in SNc (cropped blot images are shown, see Supplementary Fig. 17 for full immunoblots). Data are mean ± s.e.m.; *n* = 6 biologically independent animals. (**C**) Representative immunoblots of TH and DAT and β-actin and quantification of TH and DAT protein levels at the doses of 2, 20, 200 and 2000 μg/kg in STR (cropped blot images are shown, see Supplementary Fig. 17 for full immunoblots). Data are mean ± s.e.m.; *n* = 6 biologically independent animals; **P* < 0.05, ***P* < 0.01, and ****P* < 0.001. (**D**) Results of animals on the beam transversal, pole test, hind-climb clasping reflex and rotarod test at the doses of 2, 20, 200 and 2000 μg/kg. Data are mean ± s.e.m.; *n* = 8 biologically independent animals. One-way ANOVA was used to test for the statistical significance followed by Bonferroni’s post hoc. * *P* < 0.05, ** *P* < 0.01, and *** *P* < 0.001.

**Supplementary Fig. 2 Neuroprotective role of withaferin A is time-dependent.** (**A**) Representative immunoblots of TH and DAT and β-actin and quantification of TH and DAT protein levels during the courses of treatment at 7, 14, 21 days in SNc (cropped blot images are shown, see Supplementary Fig. 17 for full immunoblots). Data are mean ± s.e.m.; *n* = 6 biologically independent animals. (**B**) Representative immunoblots of TH and DAT and β-actin and quantification of TH and DAT protein levels during the courses of treatment at 7, 14, 21 days in STR (cropped blot images are shown, see Supplementary Fig. 17 for full immunoblots). Data are mean ± s.e.m.; *n* = 6 biologically independent animals. (**C**) Results of animals on the beam transversal, pole test, hind-climb clasping reflex and rotarod during the courses of treatment at 7, 14, 21 days. Data are mean ± s.e.m.; *n* = 9 biologically independent animals. One-way ANOVA was used to test for the statistical significance followed by Bonferroni’s post hoc. * *P* < 0.05, ** *P* < 0.01, and *** *P* < 0.001.

**Supplementary Fig. 3 Withaferin A treatment mitigates neuroinflammation in MPTP treated mice.** (**A**) Representative immunohistochemical images of GFAP and Iba1 in SNc. Scale bar, 400 μm for low-magnification and 40 μm for high-magnification images. (**B**) Representative immunohistochemical images of GFAP and Iba1 in STR. Scale bar, 400 μm for low-magnification and 40 μm for high-magnification images. (**C**) Quantification of GFAP and Iba-1 positive cells in SNc. Data are mean ± s.e.m.; *n* = 9 biologically independent animals; **P* < 0.05, ***P* < 0.01, and ****P* < 0.001 by one-way ANOVA with Bonferroni’s post hoc test. (**D**) Quantification of GFAP and Iba-1 positive cells in the STR. Data are mean ± s.e.m.; *n* = 9 biologically independent animals; **P* < 0.05, ***P* < 0.01, and ****P* < 0.001 by one-way ANOVA with Bonferroni’s post hoc test. (**E**) Representative immunoblots of GFAP, Iba1 and β-actin in SNc (cropped blot images are shown, see Supplementary Fig. 17 for full immunoblots). (**F**) Quantification of GFAP and Iba1 levels in SNc. Data are mean ± s.e.m.; *n* = 9 biologically independent animals; **P* < 0.05, ***P* < 0.01, and ****P* < 0.001 by one-way ANOVA with Bonferroni’s post hoc test.

**Supplementary Fig. 4 Activation of DJ1 by Withaferin A is dopaminergic neuron-mediated.** (**A**) Representative double immunostaining of DJ1 with TH in SNc. Scale bar, 10 μm. White arrow head indicates co-localization. (**B**) Representative double immunostaining of DJ1 with GFAP in SNc. Scale bar, 10 μm. White arrow head indicates co-localization.

**Supplementary Fig. 5 Withaferin A has little effect on DJ1 level in microglia.** (**A**) Representative double immunostaining of DJ1 with Iba1 in SNc. Scale bar, 10 μm. White arrow head indicates co-localization. (**B**) Quantification of co-localization of DJ1 with TH, GFAP or Iba1. Data are mean ± s.e.m.; *n* = 30 brain slices from 9 biologically independent animals; **P* < 0.05, ***P* < 0.01, ****P* < 0.001 and ns, not significantly by one-way ANOVA with Bonferroni’s post hoc test.

**Supplementary Fig. 6 Activation of Nrf2 by Withaferin A is dopaminergic neuron-mediated.** (**A**) Representative double immunostaining of Nrf2 with TH in SNc. Scale bar, 10 μm. **b** Representative double immunostaining of Nrf2 with GFAP in SNc. Scale bar, 10 μm. White arrow head indicates co-localization.

**Supplementary Fig. 7 Withaferin A has little effect on Nrf2 level in microglia.** (**A**) Representative double immunostaining of Nrf2 with Iba1 in SNc. Scale bar, 10 μm. White arrow head indicates co-localization. (**B**) Quantification of co-localization of Nrf2 with TH, GFAP or Iba1. Data are mean ± s.e.m.; *n* = 30 brain slices from 9 biologically independent animals; **P* < 0.05, ***P* < 0.01, ****P* < 0.001 and ns, not significantly by one-way ANOVA with Bonferroni’s post hoc test. (**C**) Quantification of nuclear translocation of Nrf2 in dopaminergic neurons of SNc. Data are mean ± s.e.m.; *n* = 30 brain slices from 9 biologically independent animals; **P* < 0.05, ***P* < 0.01 and ****P* < 0.001 by one-way ANOVA with Bonferroni’s post hoc test.

**Supplementary Fig. 8 Beneficial effect of Withaferin A on PD is STING dependent.** Representative photomicrographs and quantification of TH, GFAP, Iba1 staining in STR of STING^gt/gt^ mice, scale bar, 2 mm (upper panel) and 400 μm (down panel); *n* = 9 biologically independent animals. Data are mean ± s.e.m.; *n* = 9 biologically independent animals; **P* < 0.05, ***P* < 0.01 and ****P* < 0.001 by two-way ANOVAs followed by Tukey’s multiple comparisons test.

**Supplementary Fig. 9 Enhancement of motor function by Withaferin A in PD is STING dependent.** (**A**) Representative photomicrographs and quantification of TH, GFAP, Iba1 staining in SNc and STR of STING-KO mice, scale bar, 2 mm (upper panel) and 400 μm (down panel); *n* = 9 biologically independent animals. (**B**) Time to traverse beam apparatus, time to descend pole, hind-limb clasping reflex score, fall latency from an accelerating rotarod. Data are mean ± s.e.m.; *n* = 9 biologically independent animals; **P* < 0.05, ***P* < 0.01 and ****P* < 0.001 by two-way ANOVAs followed by Tukey’s multiple comparisons test.

**Supplementary Fig. 10 Suppression of STING by Withaferin A is dopaminergic neuron-mediated.** (**A**) Representative double immunostaining of STING with TH in SNc. Scale bar, 10 μm. (**B**) Representative double immunostaining of STING with GFAP in SNc. Scale bar, 10 μm. White arrow head indicates co-localization.

**Supplementary Fig. 11 Withaferin A has little effect on the suppression of Nrf2 level in microglia.** (**A**) Representative double immunostaining of STING with Iba1 in SNc. Scale bar, 10 μm. White arrow head indicates co-localization. (**B**) Quantification of co-localization of STING with TH, GFAP or Iba1. Data are mean ± s.e.m.; *n* = 30 brain slices from 9 biologically independent animals; **P* < 0.05, ***P* < 0.01, ****P* < 0.001 and ns, not significantly by one-way ANOVA with Bonferroni’s post hoc test.

**Supplementary Fig. 12 Withaferin A protects against MPP^+^ toxicity through DJ1-Nrf2-STING axis in human dopaminergic SH-SY5Y cells.** (**A**) The protein levels of DJ1, Nrf2, STING were analyzed by immunoblots in vector or siDJ1 knockdown SH-SY5Y cells. Data are mean ± s.e.m.; *n* = 10 from three independent experiments; **P* < 0.05, ***P* < 0.01, ****P* < 0.001 and ns, not significantly by two-way ANOVAs followed by Tukey’s multiple comparisons test. (**B**) The protein levels of DJ1, Nrf2, STING were analyzed by immunoblots in vector or siNrf2 knockdown SH-SY5Y cells. Data are mean ± s.e.m.; *n* = 10 from three independent experiments; **P* < 0.05, ***P* < 0.01, ****P* < 0.001 and ns, not significantly by two-way ANOVAs followed by Tukey’s multiple comparisons test. (**C**) The protein levels of DJ1, Nrf2, STING were analyzed by immunoblots in vector or siSTING knockdown SH-SY5Y cells. Data are mean ± s.e.m.; *n* = 10 from three independent experiments; **P* < 0.05, ***P* < 0.01, ****P* < 0.001 and ns, not significantly by two-way ANOVAs followed by Tukey’s multiple comparisons test. (**D**) Representative immunostaining of DJ1 in MPP^+^ treated SH-SY5Y cells administrated with WA or vehicle, and the quantification of immunostaining. Data are mean ± s.e.m.; *n* = 30 from four independent experiments. (**E**) Representative immunostaining of Nrf2 in MPP^+^ treated SH-SY5Y cells administrated with WA or vehicle, and the quantification of immunostaining. Data are mean ± s.e.m.; *n* = 30 from four independent experiments. (**F**) Representative immunostaining of STING in MPP^+^ treated SH-SY5Y cells administrated with WA or vehicle, and the quantification of immunostaining. Data are mean ± s.e.m.; *n* = 30, from four independent experiments. Two-way ANOVA was used to test for the statistical significance, followed by Tukey’s multiple comparisons test for multiple group comparisons. **p* < 0.05, ***p* < 0.01, and ****p* < 0.001.

**Supplementary Fig. 13 Withaferin A protects against loss of dopaminergic neurons in human α-syn overexpressing mice.** (**A**) Representational diagram of AAV injection site in mice. Briefly, 4 days after stereotactical injection of AAV-α-syn-GFP, mice intraperitoneally (i.p.) received Withaferin A (WA) (20 μg/kg) or vehicle (DMSO) per day for 90 days, tissues were harvested for molecular analyses at day 90 after the last behavior test. (**B**) Representative photomicrographs of TH staining in SNc or STR of mice stereotactically injected with vector (left) or virus (right). Scale bar, 2 mm. (**C**) Co-labelling of TH with human-α-syn showed that human-α-syn mainly expressed within the SNc dopaminergic neurons. (**D**) Diagram of the experimental design. (**E**) Representative TH staining of SNc dopaminergic neurons in human α-syn over-expressing mice treated with WA. Scale bar 400 μm. (**F**) Quantification of the SNc dopaminergic neurons in human α-syn over-expressing mice treated with WA. Data are mean ± s.e.m.; *n* = 9 biologically independent animals; **P* < 0.05, ***P* < 0.01 and ****P* < 0.001 by one-way ANOVA with Bonferroni’s post hoc test. (**G**) Representative p-α-syn immunostaining in the SNc dopaminergic neurons, scale bar 200 μm for low- magnification and 50 μm for high-magnification. (**H**) Quantification of p-α-syn levels. Data are mean ± s.e.m.; *n* = 9 biologically independent animals; **P* < 0.05, ***P* < 0.01 and ****P* < 0.001; unpaired two tailed Student’s t-tests were used for statistical analyses. (**I**) Representative immunoblots of TH, DAT, Beclin1 and P62 in SNc (cropped blot images are shown, see Supplementary Fig. 17 for full immunoblots). (**J**) Quantification of TH, DAT, Beclin1 and P62 protein levels. Data are mean ± s.e.m.; *n* = 9 biologically independent animals; **P* < 0.05, ***P* < 0.01 and ****P* < 0.001 by one-way ANOVA with Bonferroni’s post hoc test. (**K**) Representative immunoblots of α-syn, p-α-synSer129 and β-actin in detergent (Triton X-100) insoluble fractions and detergent soluble fractions in SNc of human α-syn overexpressing mice treated with WA or vehicle (cropped blot images are shown, see Supplementary Fig. 17 for full immunoblots). (**L**) Quantification of α-syn monomer, aggregation and p-α-synSer129 protein levels in detergent insoluble or soluble fractions normalized to β-actin. Data are mean ± s.e.m.; *n*= 9 biologically independent animals; **P* < 0.05, ***P* < 0.01 and ****P* < 0.001; Unpaired two-tailed Student’s t-tests were used for statistical analysis. (**M**) Relative mRNA levels of SNc. (*n* = 9 biologically independent animals). (**N**) Time to traverse beam apparatus, time to descend pole, hind-limb clasping reflex score, fall latency from an accelerating rotarod and gait analysis. Data are mean ± s.e.m.; *n* = 9 biologically independent animals; **P* < 0.05, ***P* < 0.01 and ****P* < 0.001 by one-way ANOVA with Bonferroni’s post hoc test.

**Supplementary Fig. 14 Withaferin A decreases the accumulation of p-α-syn.** Representative p-α-syn immunostaining in SNc. scale bar 200 μm, and quantification of p-α-syn levels in SNc. White arrow head indicates co-localization.

**Supplementary Fig. 15 Trans-synaptic spreading of fluorescently labelled α-syn in STR.** Stereotactic injection of human α-syn within SNc, and it’s spread to the striatum. Sagittal and coronal views are depicted in (**A**). (**B**) Representative p-α-syn immunostaining in the STR. *n* = 6 biologically independent animals.

**Supplementary Fig. 16 Original full western blot images of main manuscript.**

**Supplementary Fig. 17 Original full western blot images of supplementary figures.**
