# Supplementary figures and images for: The DJ1-Nrf2-STING axis mediates the neuroprotective effects of Withaferin A in Parkinson’s disease
Source: Cell Death Differ. 2021 Mar 24;28(8):2517–35. doi: 10.1038/s41418-021-00767-2 (PMC8329302; doi:10.1038/s41418-021-00767-2)

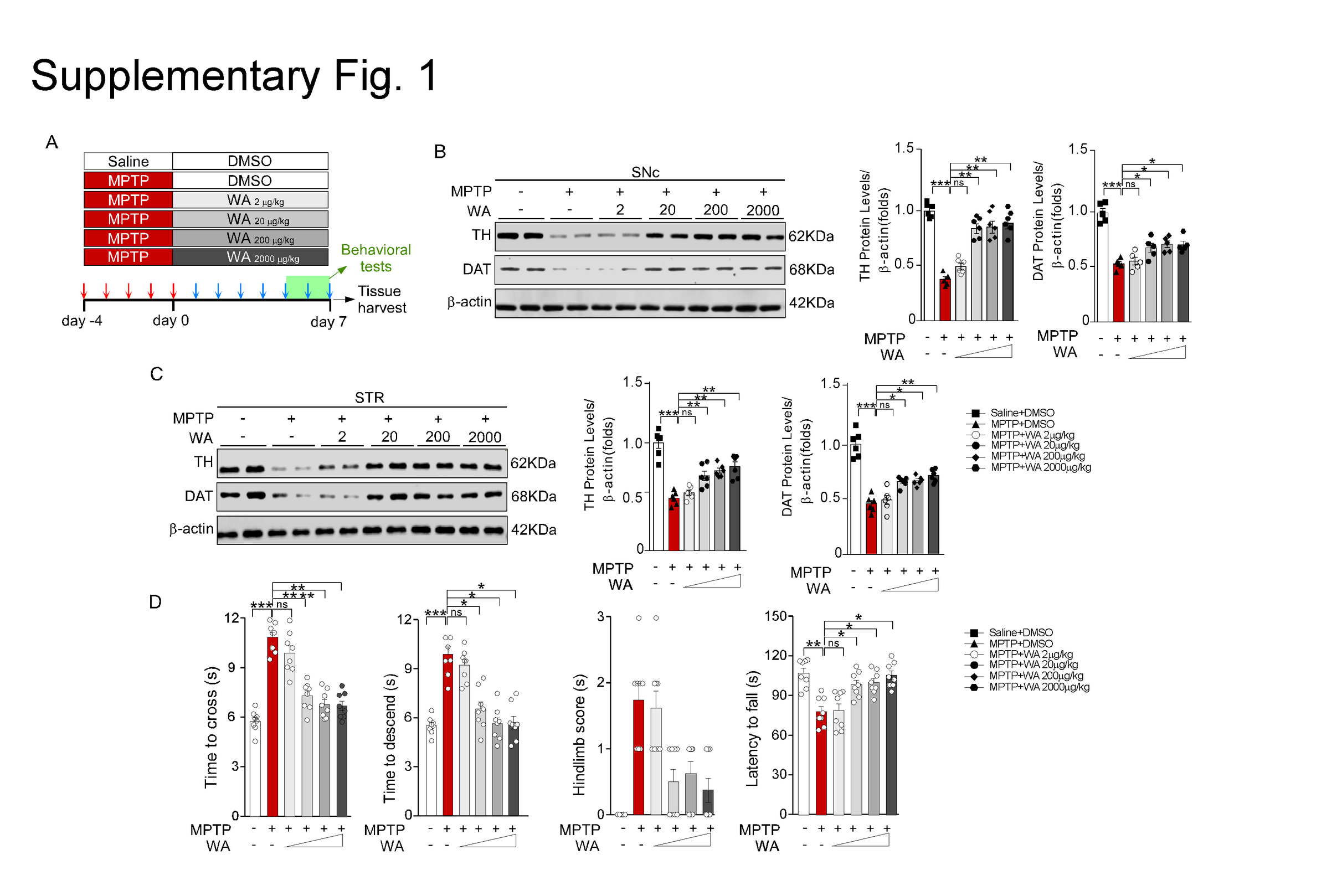

Supplement: Supplementary file 2 — Supplementary Fig 1 [file 41418_2021_767_MOESM2_ESM.tif]

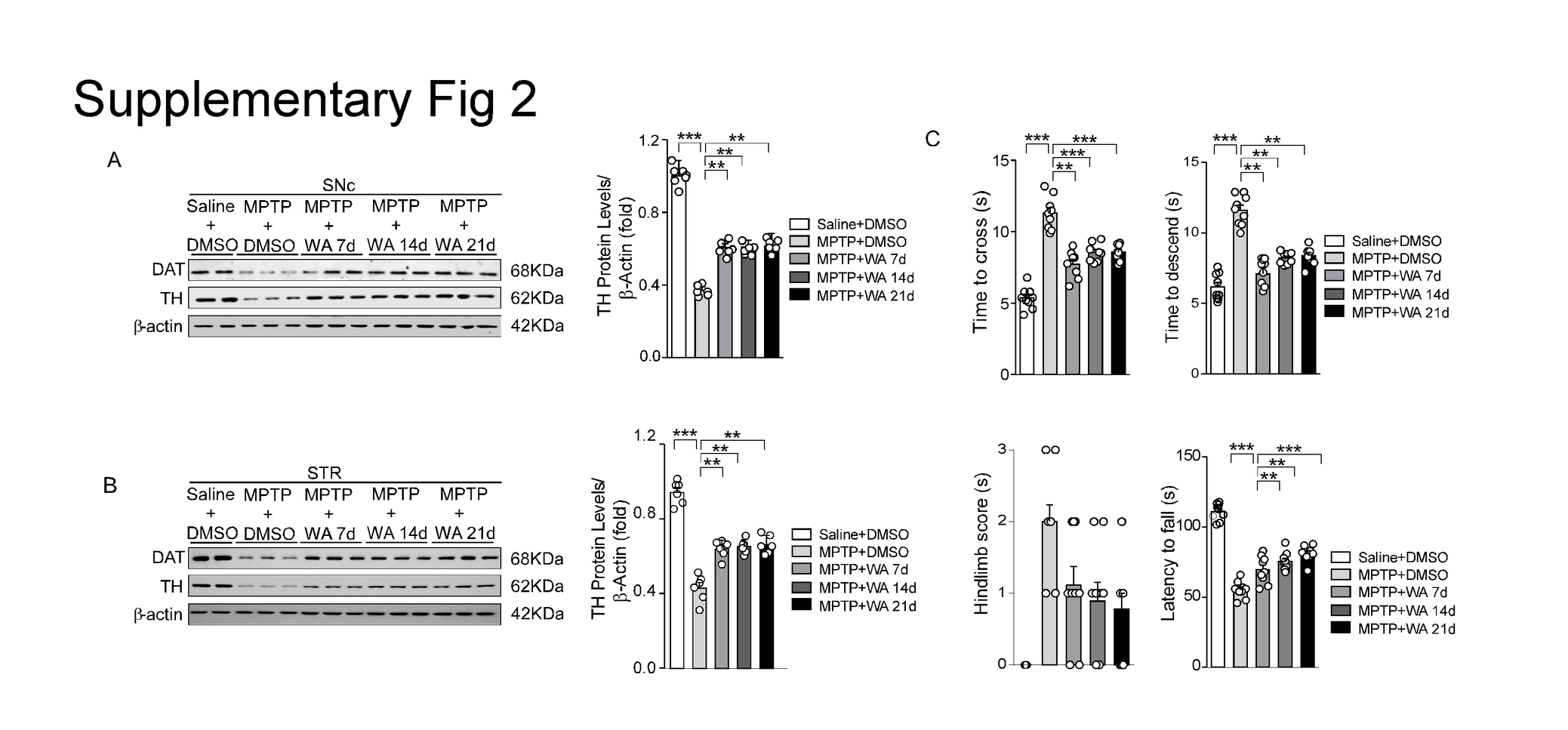

Supplement: Supplementary file 3 — Supplementary Fig 2 [file 41418_2021_767_MOESM3_ESM.tif]

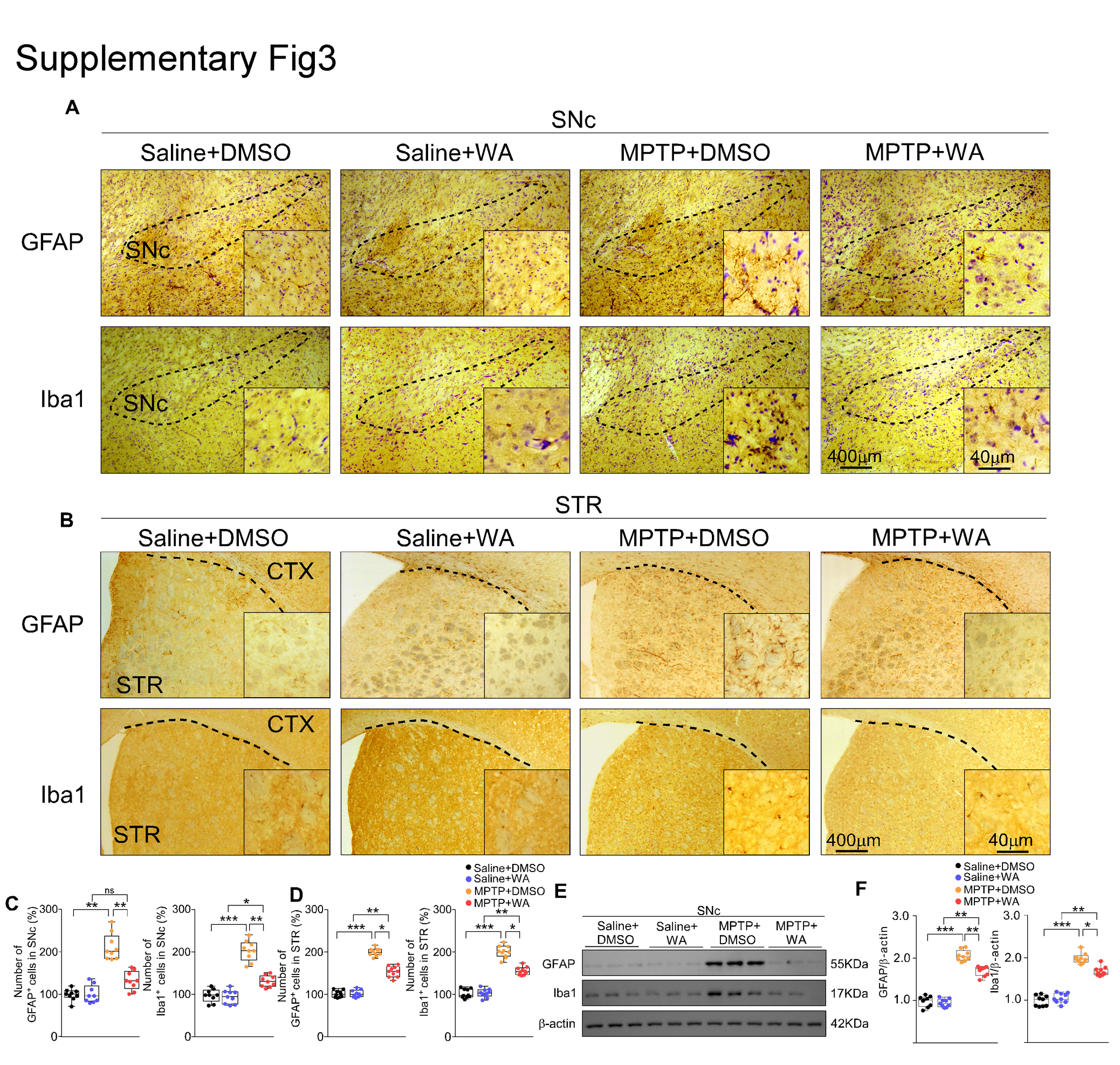

Supplement: Supplementary file 4 — Supplementary Fig 3 [file 41418_2021_767_MOESM4_ESM.tif]

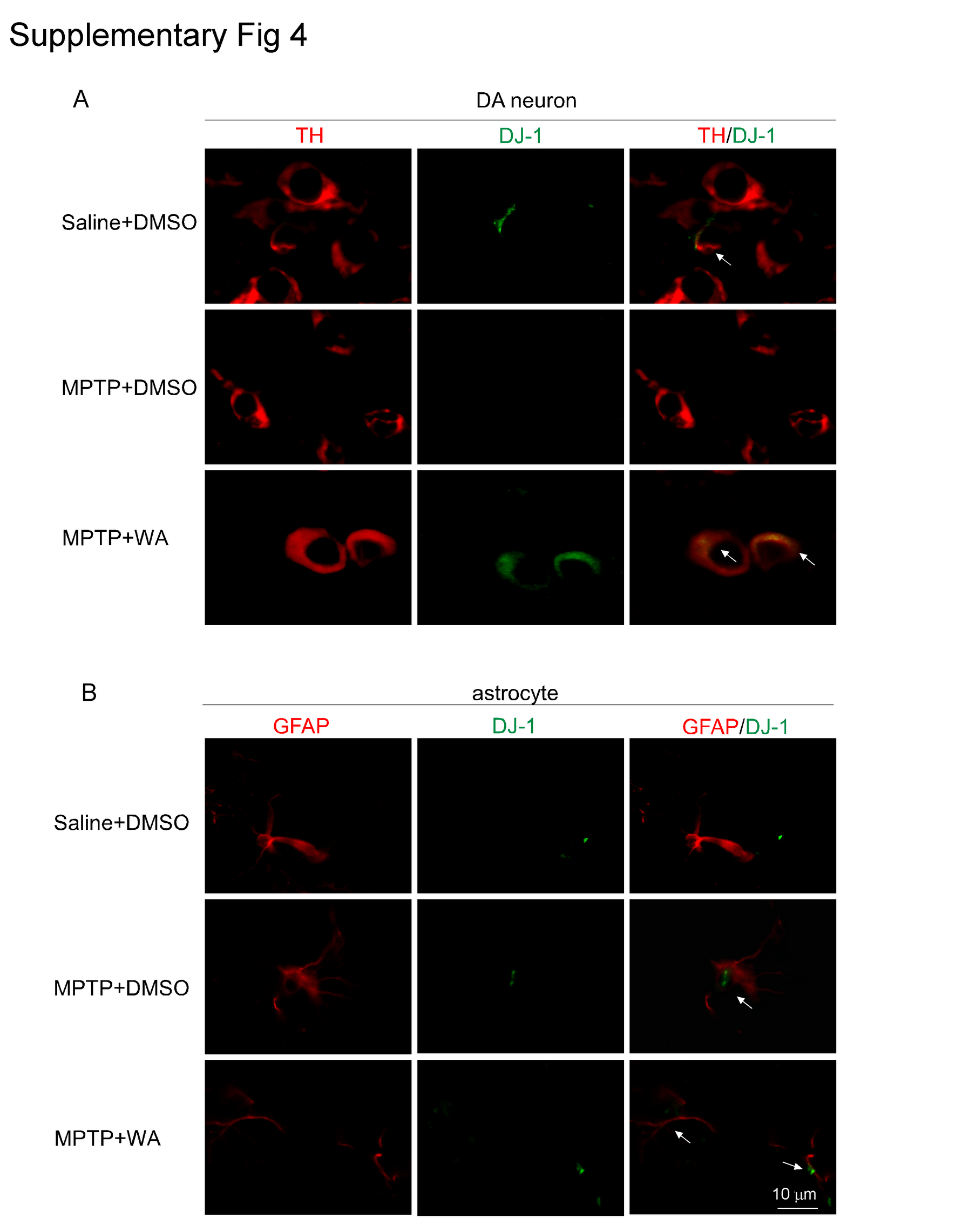

Supplement: Supplementary file 5 — Supplementary Fig 4 [file 41418_2021_767_MOESM5_ESM.tif]

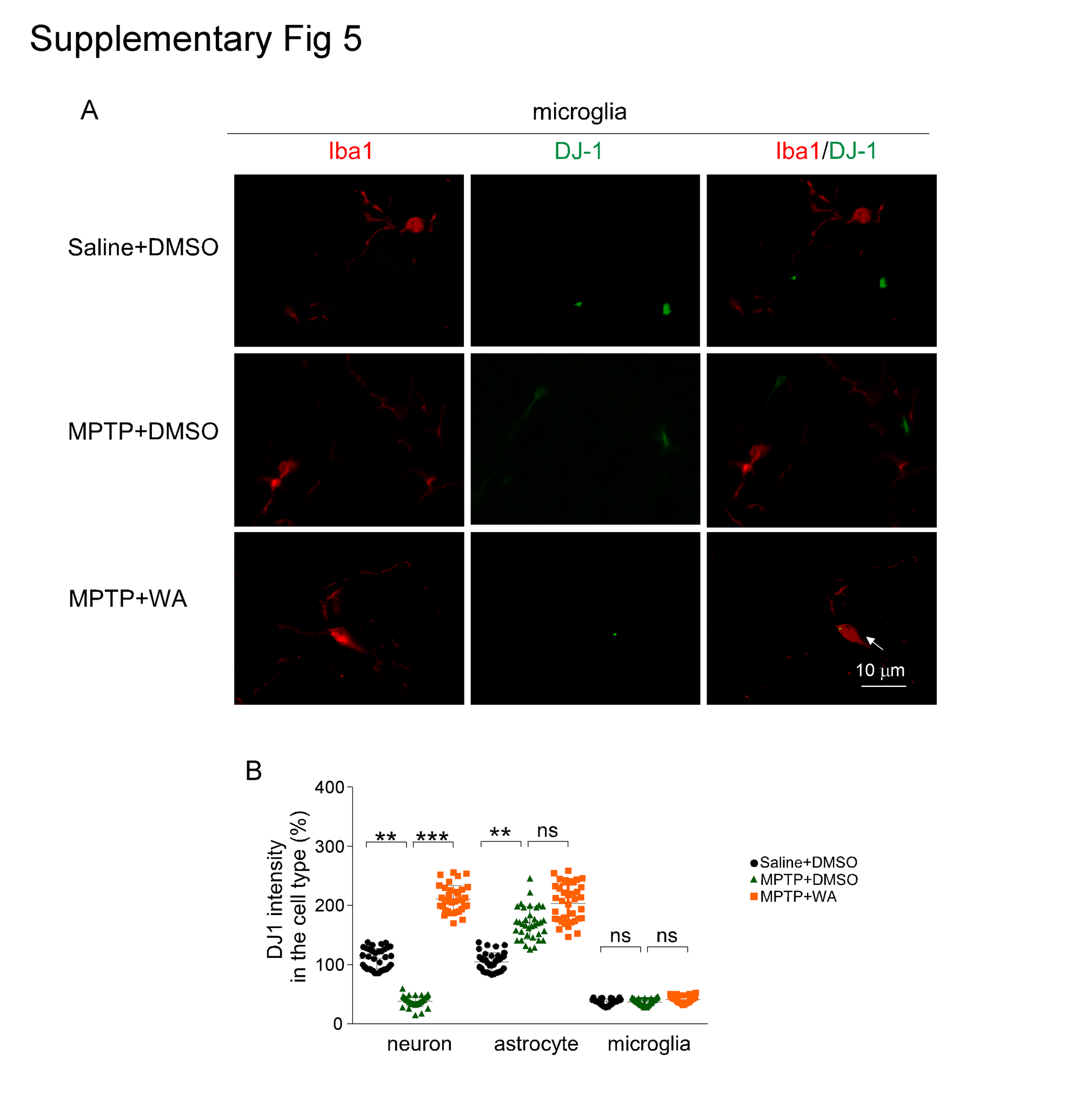

Supplement: Supplementary file 6 — Supplementary Fig 5 [file 41418_2021_767_MOESM6_ESM.tif]

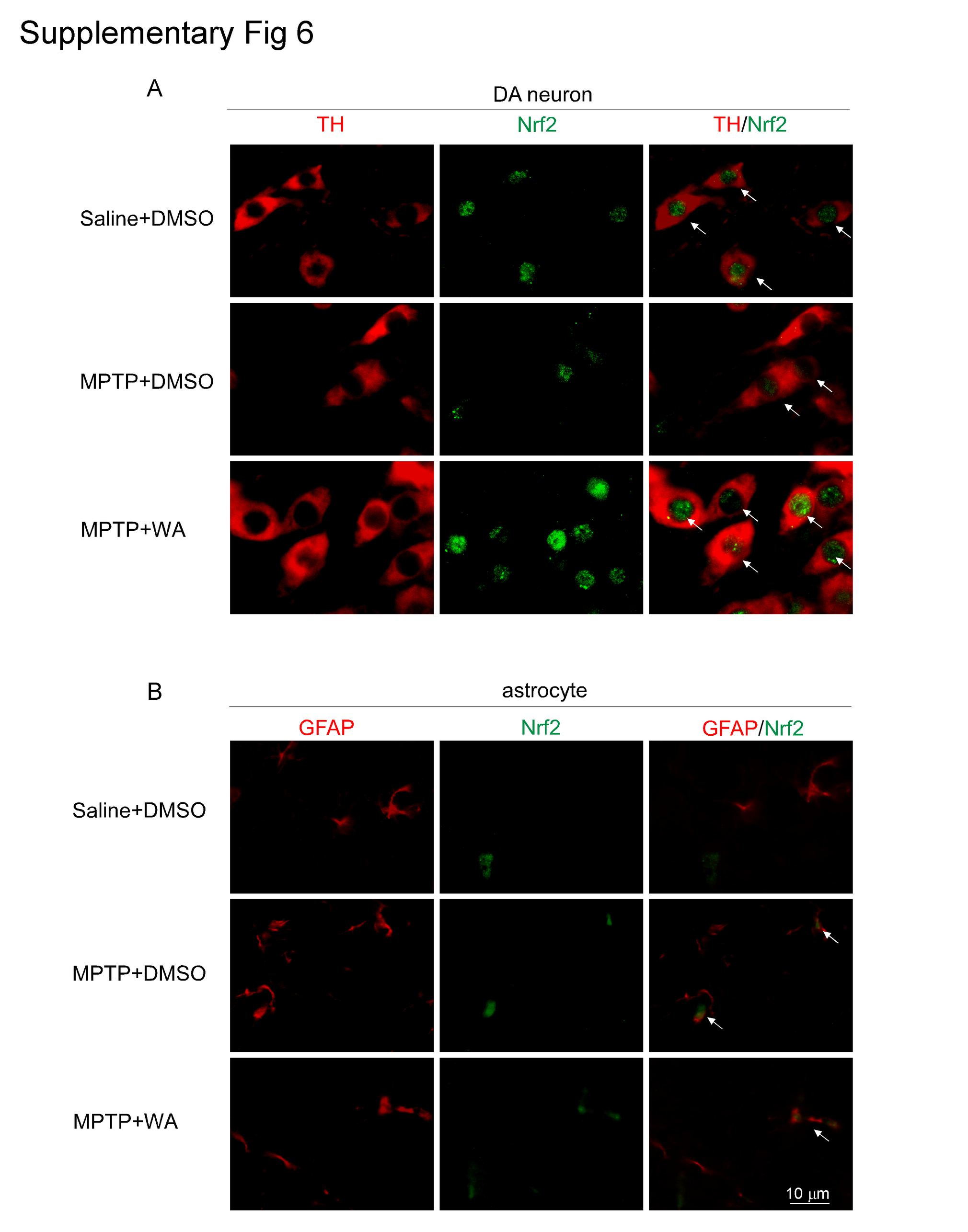

Supplement: Supplementary file 7 — Supplementary Fig 6 [file 41418_2021_767_MOESM7_ESM.tif]

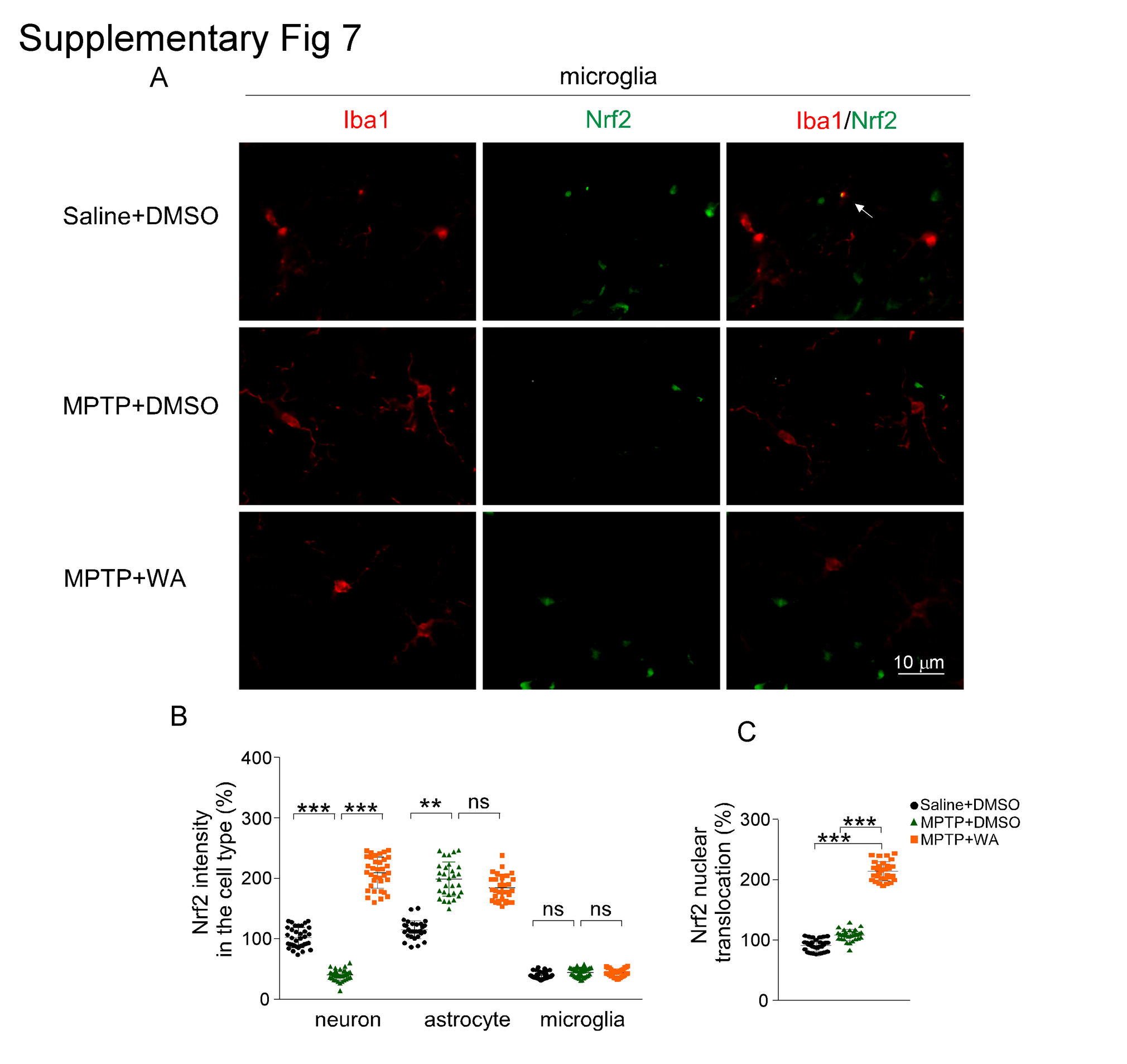

Supplement: Supplementary file 8 — Supplementary Fig 7 [file 41418_2021_767_MOESM8_ESM.tif]

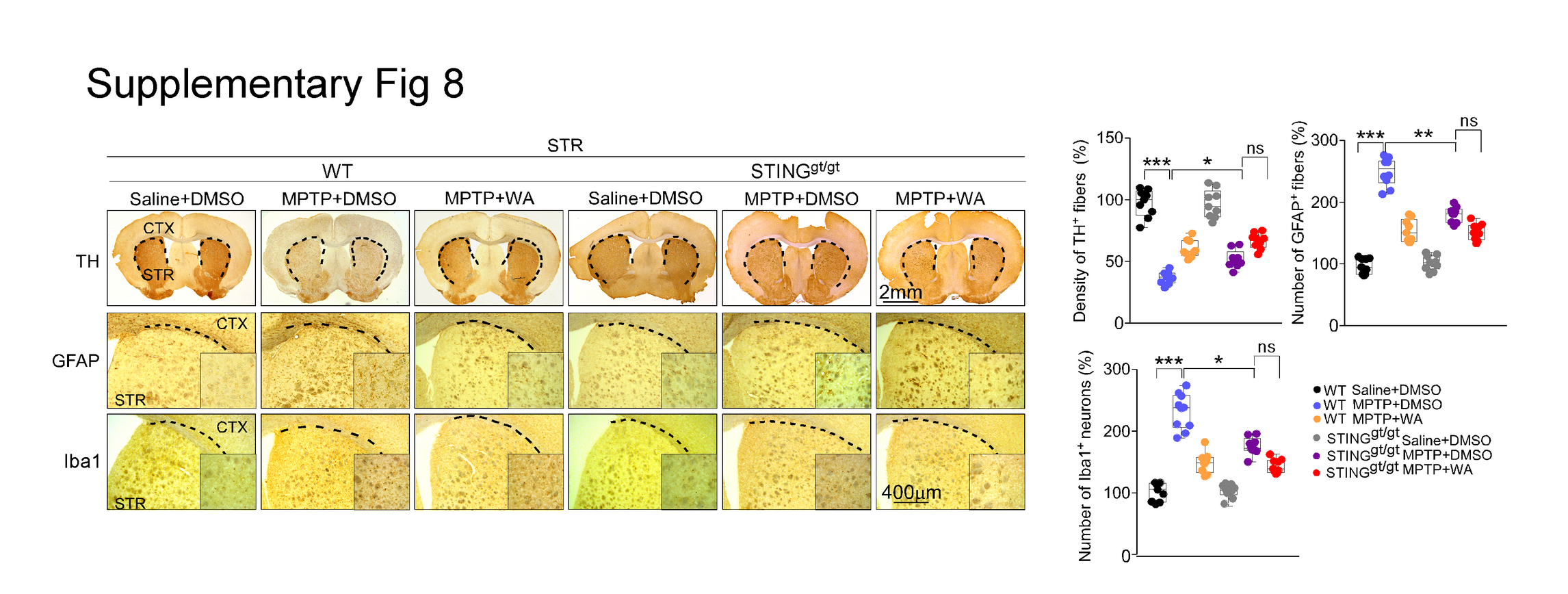

Supplement: Supplementary file 9 — Supplementary Fig 8 [file 41418_2021_767_MOESM9_ESM.tif]

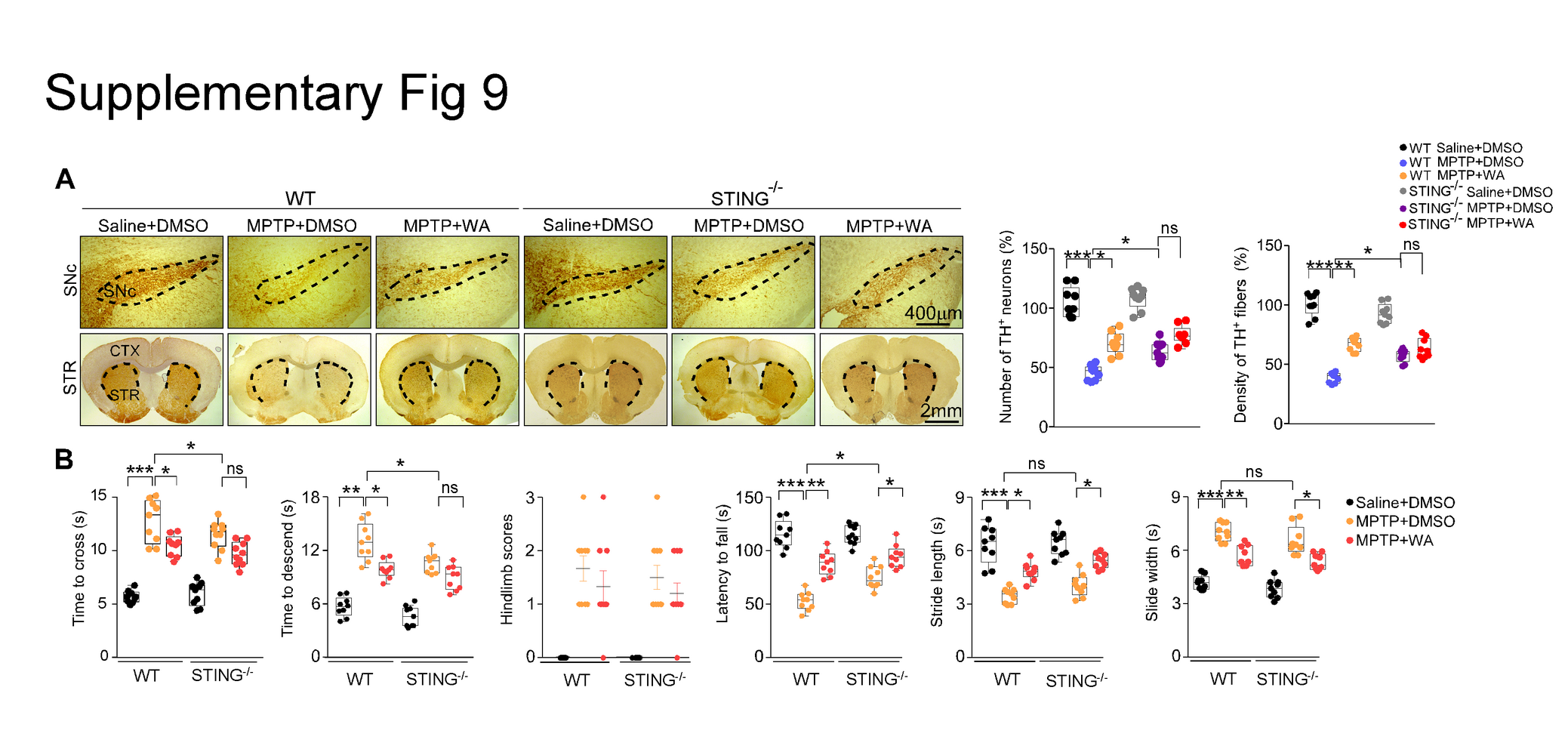

Supplement: Supplementary file 10 — Supplementary Fig 9 [file 41418_2021_767_MOESM10_ESM.tif]

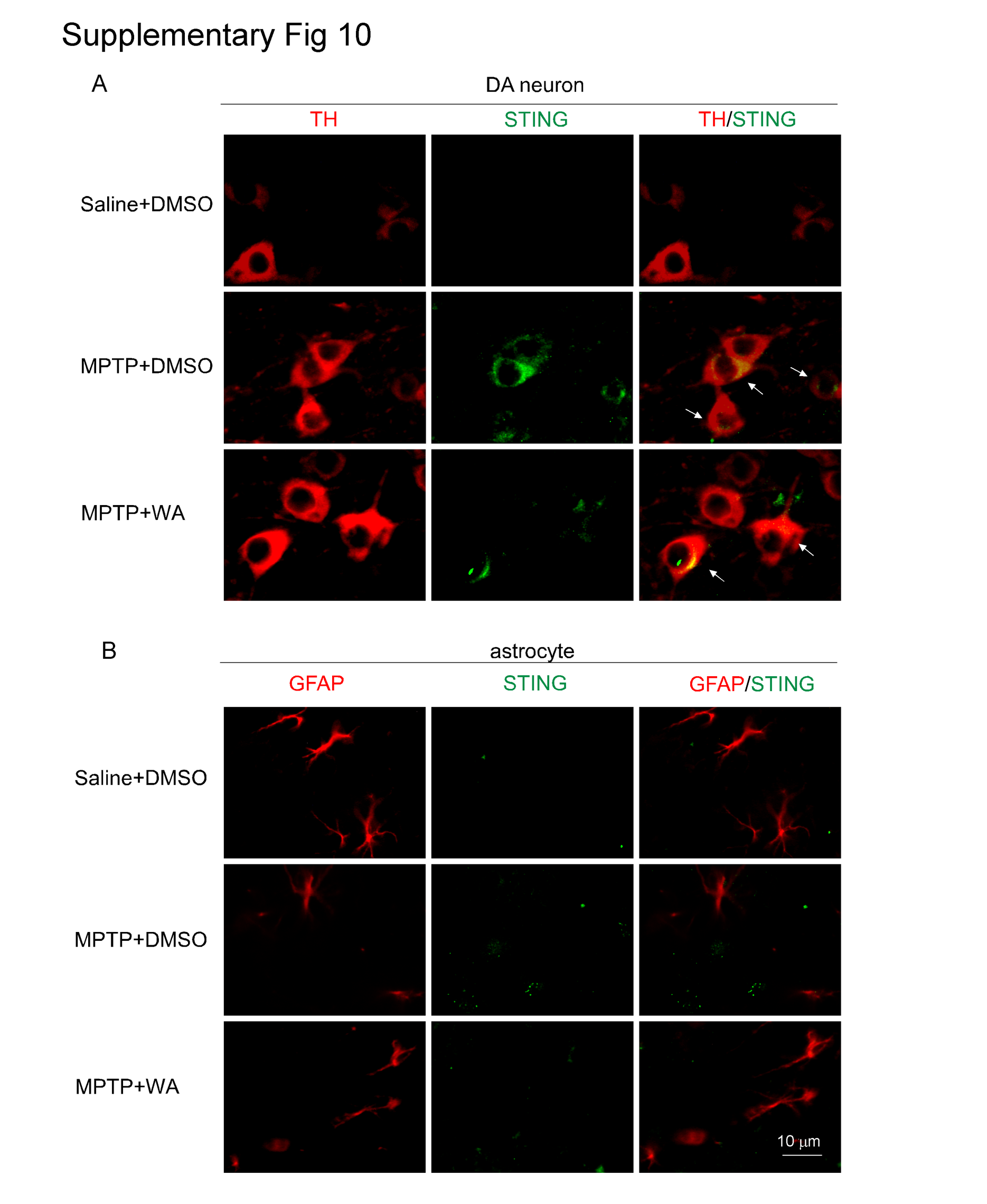

Supplement: Supplementary file 11 — Supplementary Fig 10 [file 41418_2021_767_MOESM11_ESM.tif]

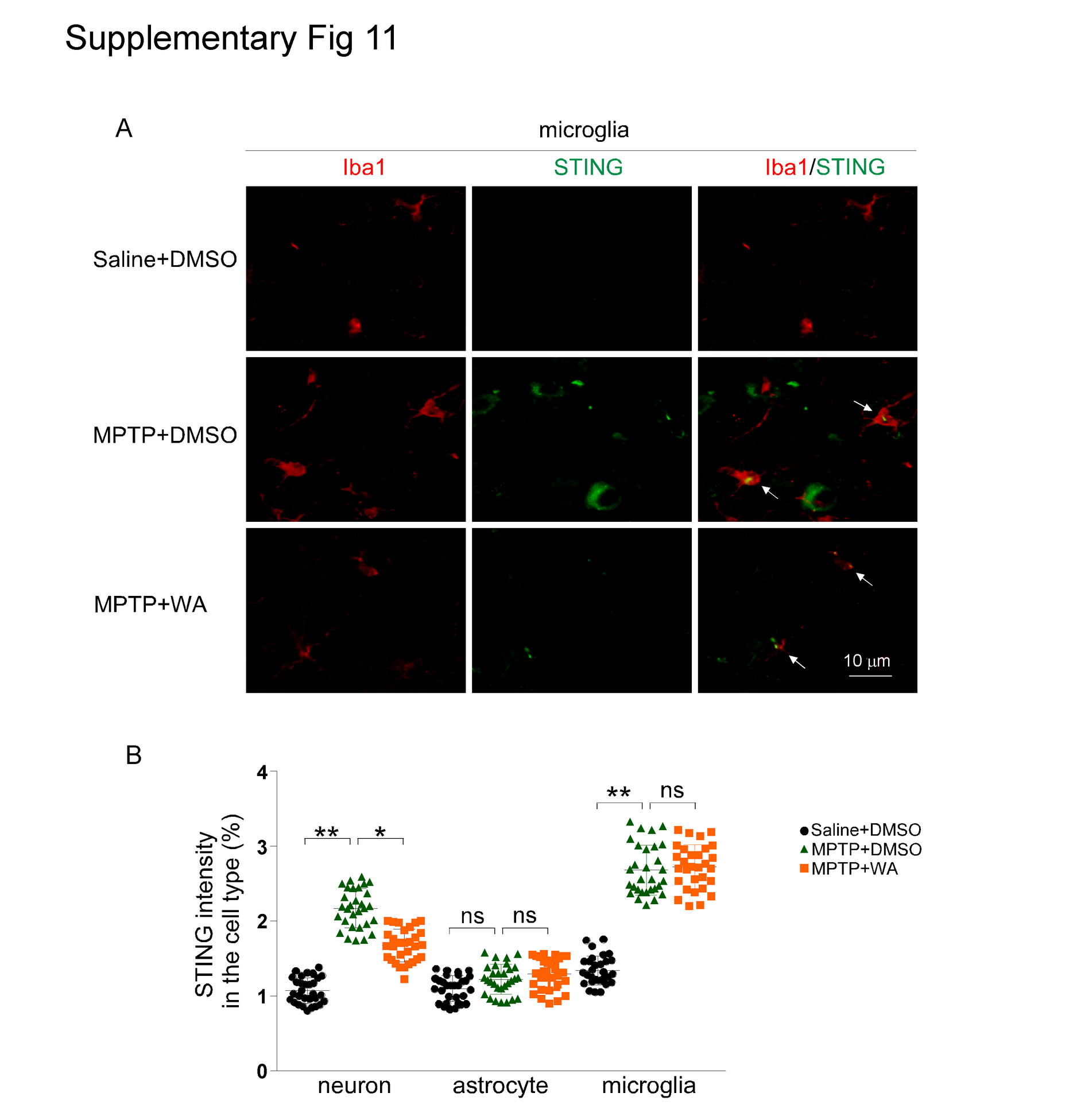

Supplement: Supplementary file 12 — Supplementary Fig 11 [file 41418_2021_767_MOESM12_ESM.tif]

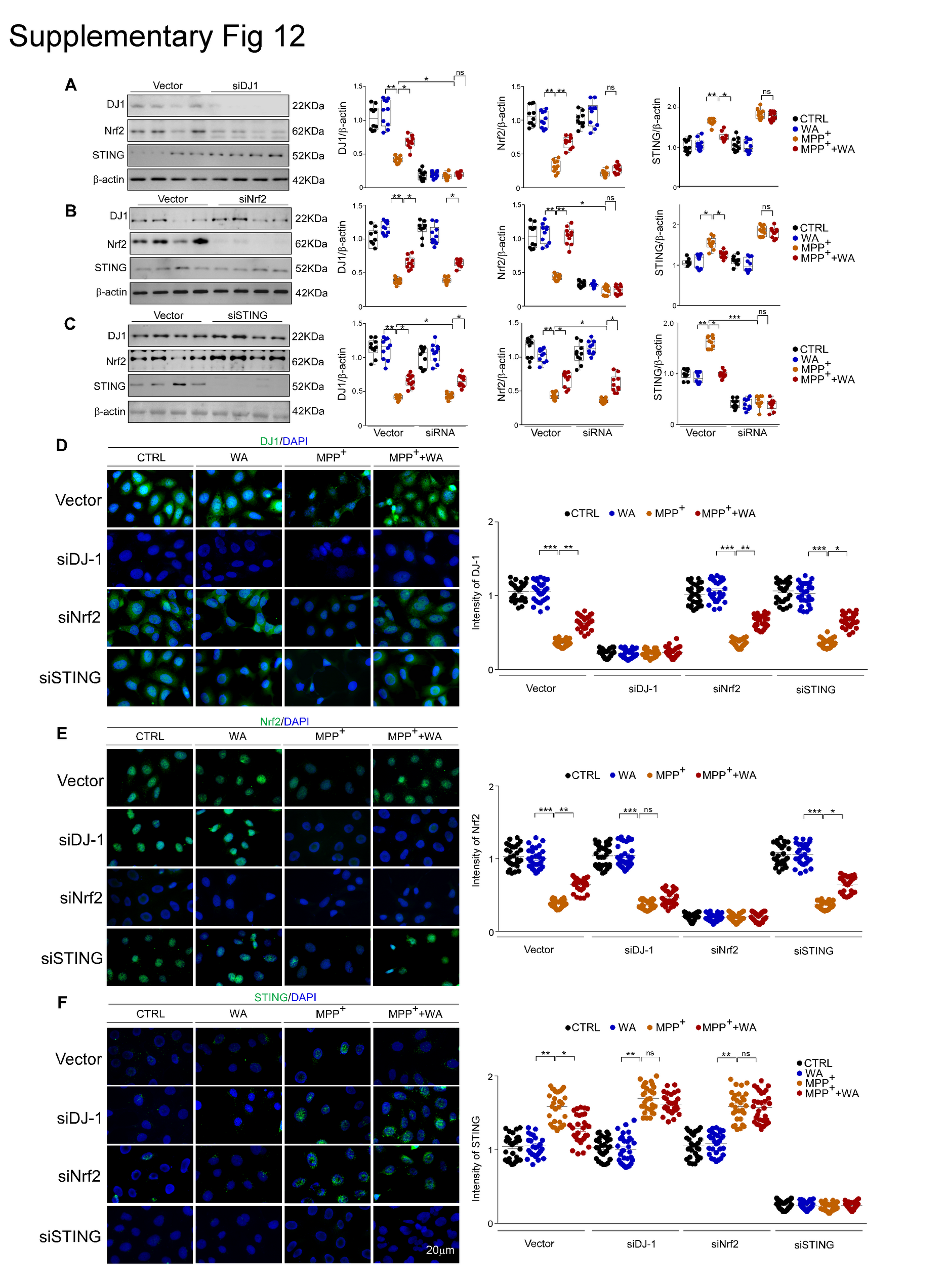

Supplement: Supplementary file 13 — Supplementary Fig 12 [file 41418_2021_767_MOESM13_ESM.tif]

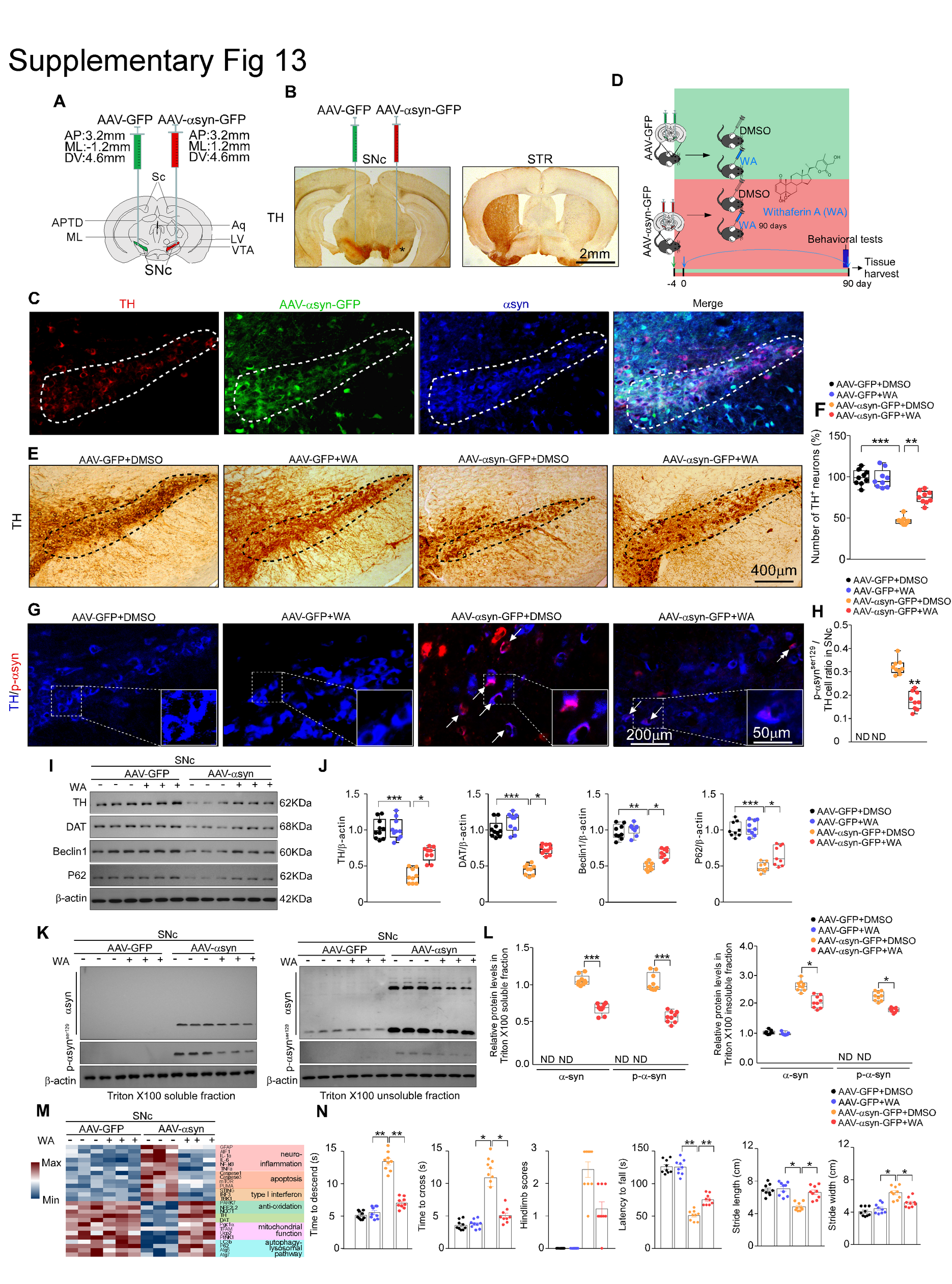

Supplement: Supplementary file 14 — Supplementary Fig 13 [file 41418_2021_767_MOESM14_ESM.tif]

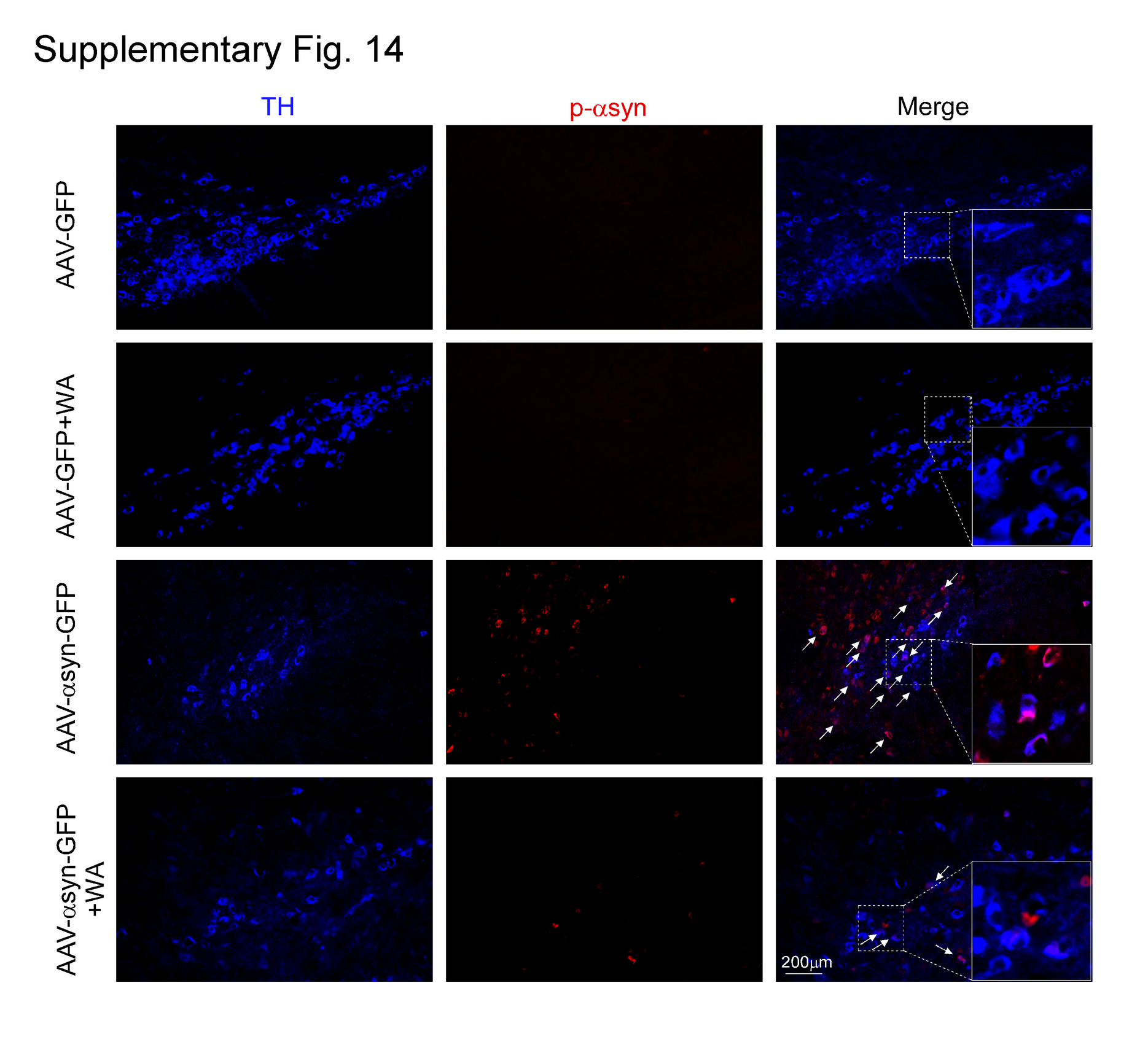

Supplement: Supplementary file 15 — Supplementary Fig 14 [file 41418_2021_767_MOESM15_ESM.tif]

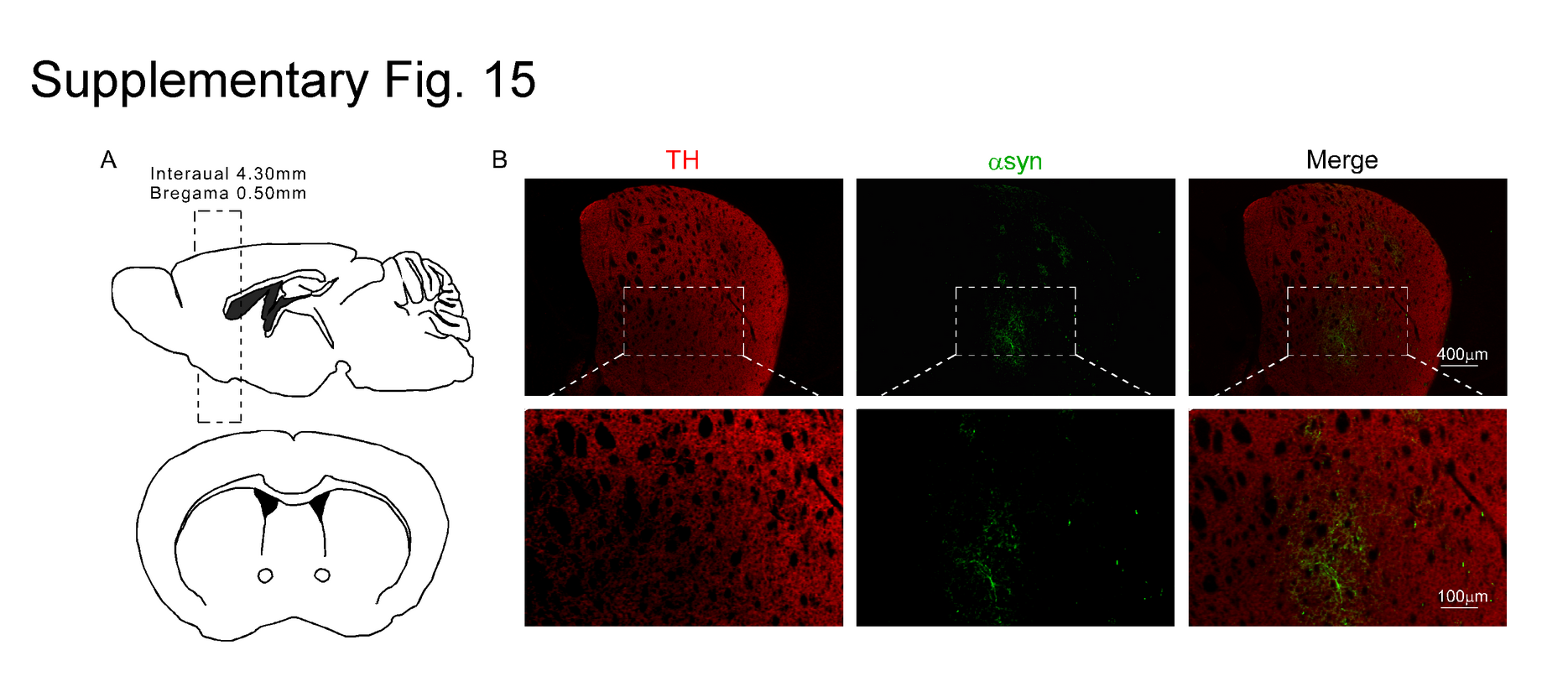

Supplement: Supplementary file 16 — Supplementary Fig 15 [file 41418_2021_767_MOESM16_ESM.tif]

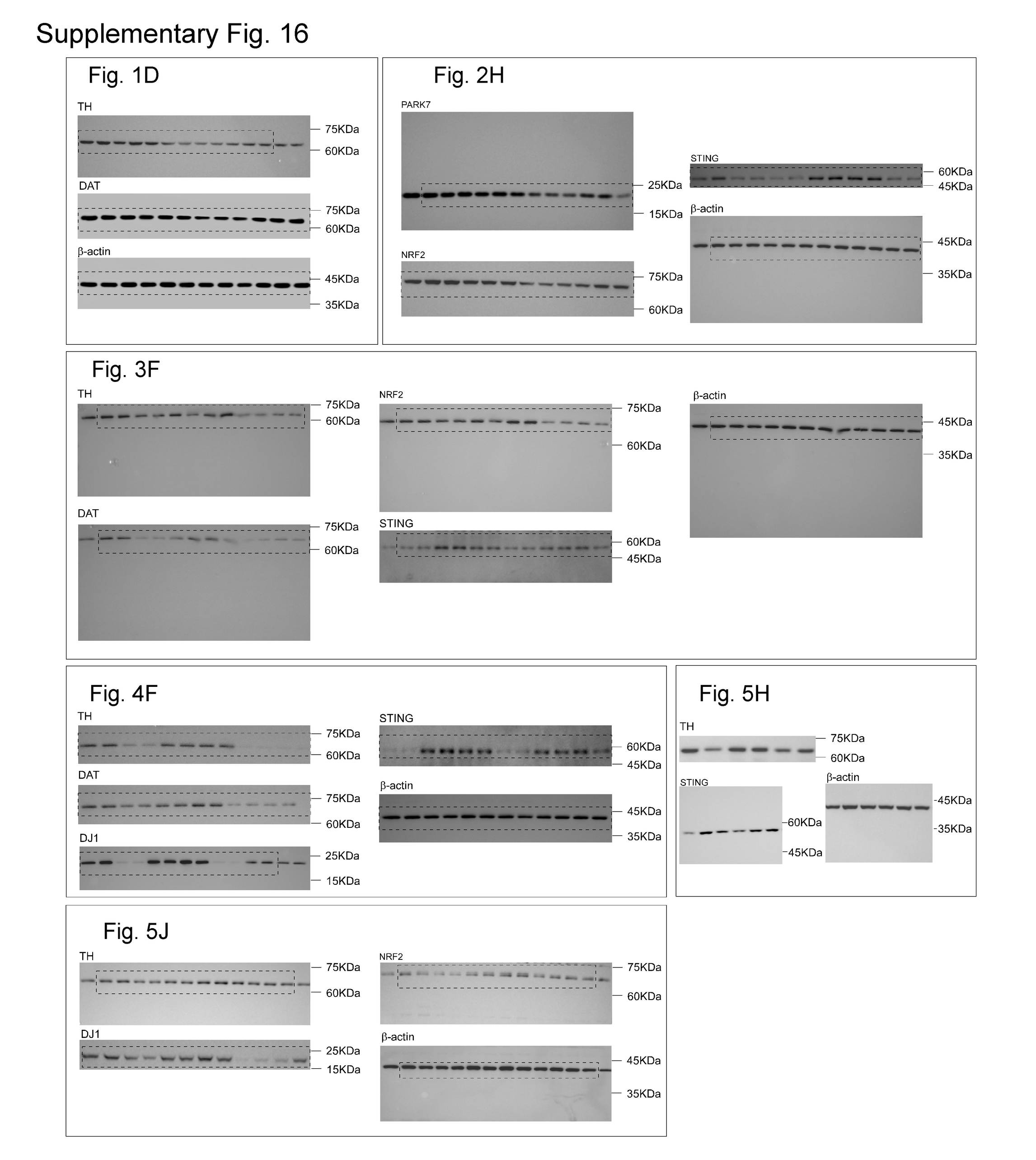

Supplement: Supplementary file 17 — Supplementary Fig 16 [file 41418_2021_767_MOESM17_ESM.tif]

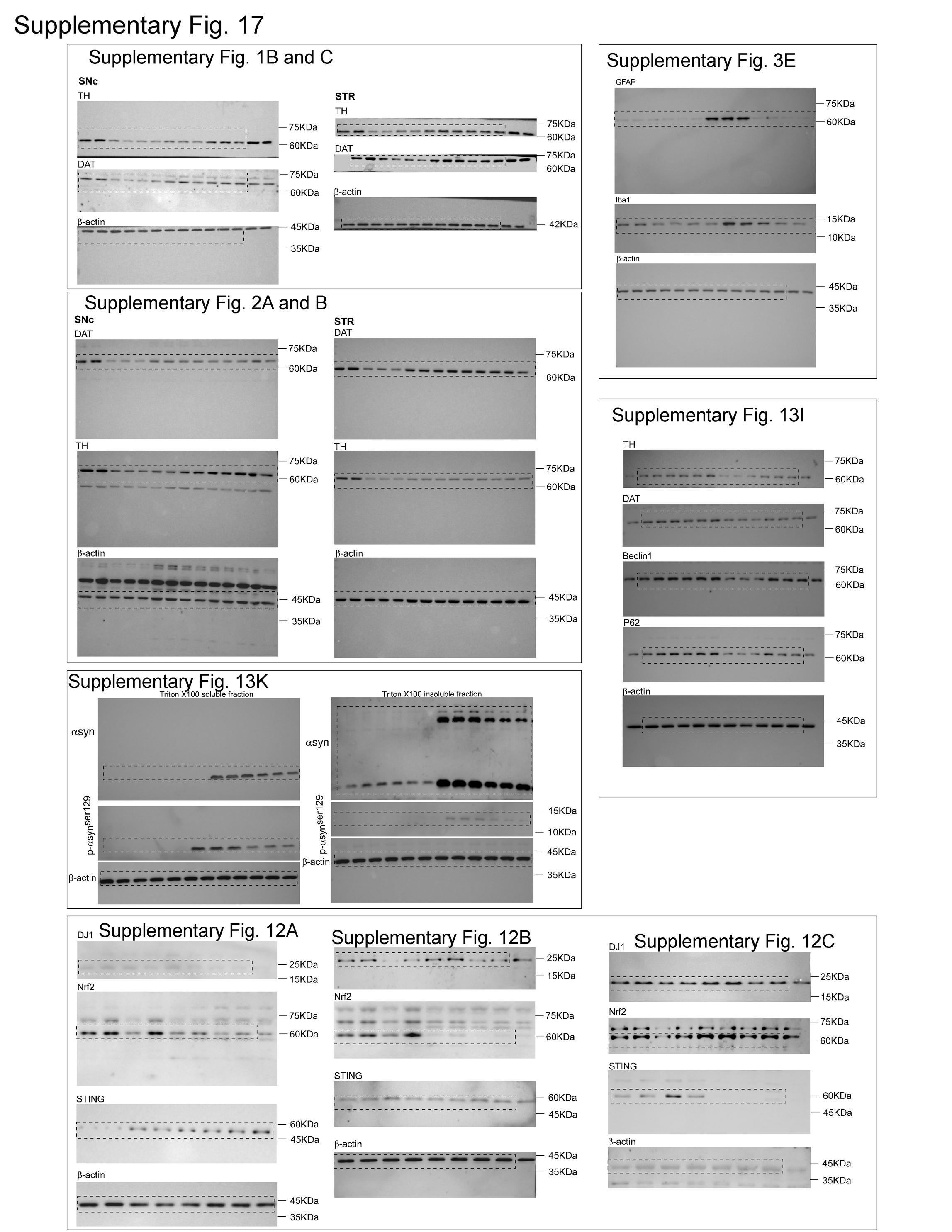

Supplement: Supplementary file 18 — Supplementary Fig 17 [file 41418_2021_767_MOESM18_ESM.tif]
