# Supplementary material for: The DJ1-Nrf2-STING axis mediates the neuroprotective effects of Withaferin A in Parkinson’s disease
Source: Cell Death Differ. 2021 Mar 24;28(8):2517–35. doi: 10.1038/s41418-021-00767-2 (PMC8329302; doi:10.1038/s41418-021-00767-2)
Supplement: Supplementary file 19 — Supplementary Tables [file 41418_2021_767_MOESM19_ESM.docx]

**Table S1: Antibodies used in this study.**

| **Antibodies** | **Source/Cat. No.** | **Host** | **Dilution** |
| --- | --- | --- | --- |
| α-synuclein | Abcam (ab138501) | Rabbit | 1:2,000 (WB)  1:500 (IHC, IF) |
| p-α-synuclein  Ser129 | Biolegend (825701) | Mouse | 1:500 (IHC, IF)  1:2000(WB) |
| Tyrosine Hydroxylase (TH) | Millipore (AB152)  Santa Cruz(sc-25269) | Rabbit  Mouse | 1:2,000 (WB)  1:500 (IHC, IF) |
| Dopamine transporter (DAT) | Bioss (bs-1714R) | Rabbit | 1:1,000 (WB) |
| DJ1 | Cell signaling (5933)  Santa Cruz (sc-55572) | Rabbit  Mouse | 1:2,000 (WB)  1:200 (IHC, IF) |
| Nrf2 | Abcam (ab31163)  Snata Cruz(sc-365949) | Rabbit  Mouse | 1:2000 (WB)  1:500 (IHC, IF) |
| STING | Proteintech (19851-1-AP)  Proteintech (66680-1-Ig) | Rabbit  Mouse | 1:500 (IHC, IF)  1:2000 (WB) |
| GFAP | Bioss (bs-0199R)  Snata Cruz(sc-33673) | Rabbit  Mouse | 1:500 (WB)  1:200 (IHC, IF) |
| Iba1 | Wako (019-19741)  GeneTex (GTX632426-S) | Rabbit  Mouse | 1:500 (IHC)  1:1,000 (WB) |
| Beclin1 | Santa Cruz (sc-48341) | Mouse | 1:500 (WB) |
| P62 | Santa Cruz (sc-48402) | Mouse | 1:500 (WB) |
| β-actin | Abcam (ab7817) | Mouse | 1:2000 (WB) |

**Table S2: Primers used in this study.**

| **Primer** | **Forward Primer 5'-3'** | **Reverse Primer 5'-3'** |
| --- | --- | --- |
| *GFAP* | CGGAGACGCATCACCTCTG | TGGAGGAGTCATTCGAGACAA |
| *AIF1* | CTTGAAGCGAATGCTGGAGAA | GGCAGCTCGGAGATAGCTTT |
| *IL1a* | GAAATGCCACCTTTTGACAGTG | TGGATGCTCTCATCAGGACAG |
| *STING* | CGTGCAGAACTCCTGTGATAAC | GTCCACCTATGCTGGAGAAGG |
| *IRF3* | ACCCTGTCATCCCACAGAG | TGTTTGGTGGAGTCCTAAGGTC |
| *TBK1* | AGCCGTGACCACTGACAACGAG | GCTGCATGGTTCTGAGTGCTAAG |
| *NFκB* | AGAGGGGATTTCGATTCCGC | CCTGTGGGTAGGATTTCTTGTTC |
| *BCL2* | CCTGTGGATGACTGAGTACC | CCCACTCGTAGCCCCTCT |
| *Bax* | GGCGAATTGGAGATGAAC | CCGAAGTAGGAGAGGAGG |
| *Atg5* | CAGCAACCCTGATGGCACCGTGTC | GGCCTCTGATGCTTGCGTCGTCTG |
| *Atg7* | ATGACGGAGCAGCCAATGAT | TCGTCACCCTTCTTCTCTGCTT |
| *Beclin1* | TGGTGCTGACGAAGATGAAG | AGGTCGAAGATGAGCACGTT |
| *USP14* | AGAGTGGAGGCGCATGCT | GGCATCTAGGAGGAAGCTGTTC |
| *P62* | TTGTCCTGGTGTGGATCGTG | TTGGAGGCAAAGGAACAGCA |
| *HSP70* | GGCAGGCAGACGAATGTTC | TTGTCATCTACGGGCACAAAG |
| *PARK7* | ATGCTAGGTAACAAGCGAATGG | TGTCGCAGAGCGGAGTAGTAT |
| *Nrf2* | TTCTTTCAGCAGCATCCTCTCCAC | ACAGCCTTCAATAGTCCCGTCCAG |
| *HO1* | CAAGCCGAGAATGCTGAGTTCATG | GCAAGGGATGATTTCCTGCCAG |
| *NQO1* | GCGAGAAGAGCCCTGATTGTACTG | TCTCAAACCAGCCTTTCAGAATGG |
| *ASK1* | AAGAGGCGGCAACAGATT | CGGTCTTGATGACGAGGG |
